# Transcriptome‐To‐Phenome Response of Larval Eastern Oysters Under Multiple Drivers of Aragonite Undersaturation

**DOI:** 10.1002/ece3.70953

**Published:** 2025-02-11

**Authors:** Samuel J. Gurr, Shannon L. Meseck, Genevieve Bernatchez, Dylan Redman, Mark S. Dixon, Lisa Guy, Aaron MacDonald, Sheila Stiles, Katherine McFarland

**Affiliations:** ^1^ National Research Council Post‐Doctoral Associate at NOAA National Marine Fisheries Service Northeast Fisheries Science Center Milford Connecticut USA; ^2^ NOAA, National Marine Fisheries Service Northeast Fisheries Science Center Milford Connecticut USA; ^3^ Inclusive NOAA Fisheries Internship Program at NOAA National Marine Fisheries Service Northeast Fisheries Science Center Milford Connecticut USA

**Keywords:** aragonite saturation state, larval development, oyster, transcriptomics

## Abstract

Understanding how interactive environmental challenges affect marine species is critical to long‐term ecological and economic stability under global change. Marine calcifiers are thought to be vulnerable to ocean acidification (OA; elevated *p*CO_2_); active dissolution of aragonite (Ω*ar*) is associated with disrupted development, survivorship, and gene expression in bivalve larvae, resulting in an early life‐stage bottleneck. Dynamic carbonate chemistry in coastal systems emphasizes the importance of multiple stressors, e.g., warming and low salinity events may change organismal responses relative to OA alone. We exposed Eastern oyster larvae ( *Crassostrea virginica* ) to a full‐factorial experimental design using two temperatures (23°C and 27°C), salinities (17 and 27), and *p*CO_2_ levels (~700 μatm and 1850 μatm *p*CO_2_), resulting in Ω*ar* conditions 0.3–1.7. Ω*ar* reduced by low salinity, elevated *p*CO_2_, and low temperature, each slowed early development and reduced survival. Low salinity × elevated *p*CO_2_ was linked to severe Ω*ar* undersaturation (< 0.5) that suppressed expression of bicarbonate transport, biomineralization and augmented expression for ciliary locomotion, proteostasis, and histone modifiers. In isolation and under moderate Ω*ar* intensity (0.5 < Ω*ar* < 1), larvae increased transcription for osmoregulatory activity and endocytosis under low salinity, and suppressed transcription for iron metabolism under elevated *p*CO_2_. Although shell growth and survival were affected by Ω*ar* undersaturation, gene expression patterns of D‐stage oyster larvae and oyster juveniles suggests tolerance to dynamic estuarine environments. Genes and expression patterns that confer survival of postmetamorphosed oysters can improve our understanding of environmental‐organismal interactions and improve breeding programs enabling sustainable production.

## Graphical abstract

As oysters emerge in the spotlight for food security and habitat health, sensitive ‐omic features are integral for development of climate‐ready strains and selective breeding programs. Our findings offer novel insights into gene candidates affecting growth and recruitment success within systems that experience seasonal warming, freshwater effluent, and coastal acidification.

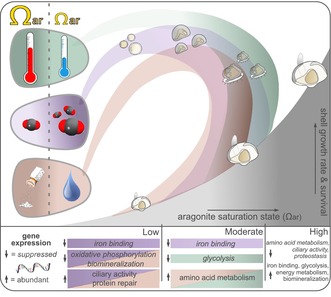

## 1 Introduction

Estuaries are highly variable environments that experience rapid fluctuations in environmental conditions (Cai et al. 2011; Ekstrom et al. 2015). Riverine discharge (i.e., freshwater effluent and rainfall; Bianucci et al. 2018; Day and Rybczyk 2019), warming from proximity to land (Scanes, Scanes, and Ross 2020), upwelling/physical advection (Gruber et al. 2012), and ecosystem microbial metabolism (Herrmann et al. 2015; Wallace, Peterson, and Gobler 2021) drive persistent and diurnal changes. Multidecadal datasets in estuaries describe an annual freshening and warming (−0.086 year^−1^ and + 0.21°C–0.34°C decade^−1^, respectively; Georgas et al. 2016; Preston 2004) and pH variability (i.e., −0.023 to +0.023 ΔpH yr.^−1^; Carstensen and Duarte 2019) occurring at a rate faster than the open ocean (~4–10×; Carstensen et al. 2018; Lowe, Bos, and Ruesink 2019). Thus, estuarine fauna are subject to dynamic variations, such that organismal responses must cope with a higher frequency and magnitude than the global average (Shen et al. 2020; Gunderson, Armstrong, and Stillman 2016).

The ecological, economical, and social‐cultural importance of bivalves has resulted in a substantial body of research describing differential susceptibility to salinity (Pourmozaffar et al. 2020), temperature, and acidification (Gazeau et al. 2013; Kroeker et al. 2013), especially during early embryonic and larval development (Gazeau et al. 2011; Kurihara 2008; Timmins‐Schiffman et al. 2013; Waldbusser et al. 2013). The Eastern oyster, *Crassostrea virginica*, possesses high phenotypic plasticity, showing tolerance to extreme variation in environmental conditions on seasonal and tidal scales. Post metamorphic oysters are euryhaline tolerating salinities < 3 for prolonged periods (McFarland et al. 2022a; Southworth, Long, and Mann 2017) and as high as 40 (Galtsoff 1964). In contrast, the pelagic larval stage is far more sensitive to such fluctuations, and changes in salinity > 10 have resulted in high mortality during laboratory exposures (McFarland et al. 2022b). Similarly, postmetamorphic oysters can tolerate seasonal temperature variation from −2°C to 36°C and internal temperatures of > 40°C during low tide emersion (Galtsoff 1964), but during early development (gametes, embryos, larvae), temperature deviations > 10°C can be lethal (McFarland et al. 2022b) and coupled osmotic and thermal challenges can increase larval sensitivity to other stressors (Ko et al. 2014; MacInnes and Calabrese 1979). While *C. virginica* larvae are more resilient to acidification than other coastal bivalves (up to 1500 μatm *p*CO_2_), larvae are sensitive to prolonged deviations in seawater chemistry (Gobler and Talmage 2014). Despite decades of research, few experiments have investigated how coupled challenges affect oyster larvae.

Reduced pH and carbonate ion levels due to absorption of atmospheric carbon dioxide (*p*CO_2_) is termed ocean acidification (OA; Cai et al. 2011; Doney et al. 2009), which has become a global concern (IPCC 2023) intensified by regional‐scale change in marine coasts (Van Dam and Wang 2019; Wallace et al. 2014). Aragonite saturation state (Ω*ar*) is commonly used to track OA because aragonite is usually the first form of crystallized carbonate that an organism produces (Jacob et al. 2008). The mineralogy and structure of bivalve shells is biologically controlled (Checa et al. 2007); however, reduced carbonate from OA may impede calcification for some marine bivalves (Kroeker et al. 2013) and delay larval development (Waldbusser et al. 2015a, 2015b). Ω*ar* is a unifying metric affected by multiple parameters (i.e., salinity, temperature, calcium, and carbonate ions) and numerous models highlight correlative patterns of Ω*ar* with temperature (Reum et al. 2014) and salinity (Bianucci et al. 2018) in estuaries, among other variables (i.e., oxygen and ecosystem heterotrophy; Borgesa and Gypensb 2010; Cotovicz Jr., Marins, and da Silva 2022; Shen et al. 2019).

At Ω*ar* < 1, thermodynamical, active dissolution may occur (Zeebe and Wolf‐Gladrow 2001). Reduction in Ω*ar* is further accelerated by seawater dilution (riverine input; Van Dam and Wang 2019) and this freshening can overlap temporally with reproduction and early development (Salisbury et al. 2008). In an already acidified coastal region, low salinity events can cause Ω*ar* to quickly fall to < 1. Warming increases Ω*ar* due to the tendency for seawater to degas CO_2_ (decrease dissolved inorganic carbon and total alkalinity, as DIC and TA), in contrast to colder temperatures that absorb more CO_2_ (increase DIC and TA), decreasing Ω*ar*. Warming could ameliorate some effects of OA though temperature only has marginal effects on Ω*ar*. Bivalve larvae reduce survival and growth at moderate Ω*ar* levels (Ω*ar* ~ 1.4–2.0; Gazeau et al. 2011; Hettinger et al. 2012; Ries et al. 2016; Talmage and Gobler 2010), however effects are predominantly studied as responses to *p*CO_2_‐driven change. Despite the correlative nature of *p*CO_2_, temperature, and salinity, multifactorial effects in the context of Ω*ar* remain poorly understood. The seasonal variability of Ω*ar* undersaturation in estuaries (Cai et al. 2021; Evans et al. 2019; Pelletier et al. 2018) demands experimental approaches that address both Ω*ar* and correlative variables.

A notable rise in transcriptomic profiling under environmental challenges (such as OA; Strader, Wong, and Hofmann 2020) aims to develop molecular markers associated with environmental tolerance and beneficial economic traits (i.e., growth, immunity, resilience, etc.; Chandhini and Rejish Kumar 2019). Molecular‐level processes occur as precursors to phenotypic effects (Shamir et al. 2016) and employ conserved cellular‐molecular networks (Kültz 2005), positing an informative and transferable tool among phyla to infer plasticity and adaptive responses. Paired with phenotypic measurements, transcriptomics has identified a collection of proteins involved in initial larval shell formation (De Wit et al. 2018), developmental timing‐by‐environment responses (Wright‐LaGreca, Mackenzie, and Green 2022), and resilience in oysters and clams (Goncalves et al. 2017a; Gurr et al. 2022). Oysters in particular display an array of inducible genes that highlight their evolutionary adaptations to extreme intertidal conditions, including elevated osmoregulatory activity (i.e., calcium‐dependent signal transduction pathway; Zhao et al. 2012), protein stabilization under low salinity (i.e., chaperones; Maynard et al. 2018), cytoprotection under thermal stress (i.e., antioxidants; Farcy et al. 2009), compensatory energy metabolism (Tomanek et al. 2011), and both raised and neutral transcriptional change to permit energetically‐expensive processes under *p*CO_2_‐driven Ω*ar* undersaturation (i.e., shell biomineralization; De Wit et al. 2018; Wright‐LaGreca, Mackenzie, and Green 2022). Under multifactorial environmental challenges, transcriptome profiling of oysters document adaptations in energy metabolism (i.e., low pH × high temperature; Chapman et al. 2011), however cytoprotection can be compromised under aggregate abiotic change (i.e., high temperature × low salinity; Ertl, O'Connor, and Elizur 2019). Thus, multifactorial experiments are critical to disentangle key environmental variables that affect phenotype and transcription.

A growing body of research suggests that co‐occurring abiotic factors elicit additive or multiplicative responses, highlighting the need for multifactorial experiments (Boyd et al. 2018; Przeslawski, Byrne, and Mellin 2015). Prolific documentation of osmotic, thermal, and OA tolerance suggests that the Eastern oyster *C. virginica* is an ideal model to understand adaptive transcriptome‐to‐phenome effects of environmental change using these three environmentally relevant, co‐occurring stressors. In this study, *C. virginica* were exposed from embryos until settlement as recently metamorphosed juveniles to low v. high salinity × *p*CO_2_ × temperature in a crossed, full‐factorial design to understand how environmental covariance affects organismal response. To this end, we used gene expression analysis to examine the transcriptional underpinnings of growth, survival, and respiration rate in response to multiple drivers of reduced Ω*ar*.

## 2 Materials and Methods

### 2.1 Experimental Design and Larval Rearing

**FIGURE 1:**
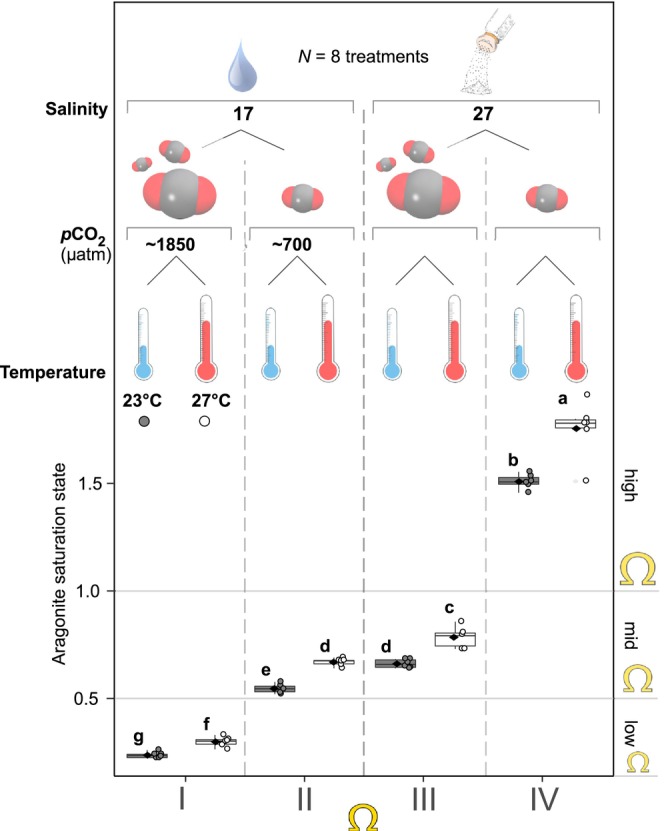
Experiment schematic for the full reciprocal challenge under high and low salinity, *p*CO_2_, and temperature (*N* = 3 replicates treatment^−1^). Boxplots present the averaged aragonite saturation values for replicates within sampling date and treatment (*N* = 6 timepoints). Letters represent Tukey's HSD post hoc effects of a one‐way ANOVA. Four treatment bins are proposed based on major effectors of reduced aragonite saturation state: (Ω*ar*‐I) severely undersaturated driven by high *p*CO_2_ and low salinity, (Ω*ar*‐II) moderately undersaturated driven largely by low salinity, and (Ω*ar*‐III) moderately undersaturated driven largely by high *p*CO_2_. Lastly, a “control” treatment (Ω*ar‐*IV) of high aragonite saturation under low *p*CO_2_ and high salinity.

Embryos were supplied by the Noank Aquaculture Cooperative in Groton, CT on April 29, 2021. Pooled gametes from 6 males and 4 females were mixed to fertilize at a salinity of 29 and a temperature of 28°C then transported to the NOAA/NMFS Milford Shellfish Laboratory in Milford, CT (41°12′44.6″N 73°03′10.6″W). Embryos were enumerated and distributed to their treatment condition within 4 h of fertilization. Treatment conditions included two OA levels (~700 μatm and ~ 1850 μatm *p*CO_2_) two temperatures (23°C and 27°C), and two salinities (17 and 27) in a full factorial design (*N* = 3 replicate tanks treatment^−1^; Figure 1). Seasonal carbonate chemistry for Long Island Sound (LIS) historically has been sporadic; however averaged seawater *p*CO_2_ collected in recent years ranges from 600 to 1000 μatm (Padilla et al. 2024; Barrett et al. 2024), and the NOAA/NMFS Milford Shellfish Laboratory has weekly records of seawater carbonate chemistry (using same methods described below) from 2020 to modern day that can exceed 1800 μatm in the summer (*unpublished data*). Larval studies on bivalve species suggest no response to conditions below 1500 μatm *p*CO_2_ (Gobler and Talmage 2014) and our pilot data on larvae *C. virginica* found no difference in survival between 730 and 1620 μatm *p*CO_2_ (*unpublished data*). Thus, we chose OA levels representative of the local average and upper extreme during summer when oysters spawn. High salinity approximated the conditions observed at the Noank Aquaculture Cooperative where adult oysters were conditioned and spawning took place, and a decrease in salinity by 10 was chosen based on McFarland et al. (2022b) to elicit a response without causing complete mortality of the treatment. Lastly, ~23°C is the ambient subtidal maximum from historical records at the experiment site (records 1960–2015; *unpublished data*) and 27°C was chosen as a + 4°C future scenario.

OA levels were achieved by continuously bubbling a mixture of CO_2_‐stripped air and research grade CO_2_ mixed at different ratios using mass flow controllers (Aalborg Instruments and Controls, Orangeburg, NY, USA) directly into static experimental chambers. All treatment water was preconditioned by bubbling for a minimum of 12 h prior to water changes. The low salinity treatment was achieved by diluting ambient seawater (~27) with deionized water. Temperatures were maintained using aquarium heaters in water baths. Salinity treatments were made prior to CO_2_ introduction and seawater was held in water tables to achieve target temperature conditions during the CO_2_ conditioning.

Initial development occurred in 20‐L buckets at a density of 30 larvae mL^−1^. Larger vessel volume and high stocking density was employed for the initial 24 h because of the expected high mortality and biomass needs for RNA extraction. After approximately 24 h postfertilization (hpf), water changes were completed, ~10,000 pooled larvae from each replicate tank were preserved in RNAlater (Thermo Fisher Scientific) for RNA extraction, and survival to D stage (the first shelled stage) was calculated for each replicate. Sampling estimates for molecular extraction were based on volumetric counts after animals were screened and condensed to a small volume of ~25–50 mL filtered seawater. Survival counts served only to estimate survivorship during this critical 24‐h developmental period and stands alone from all other assessments during the experiment. D‐hinge larvae were divided into 3‐L chambers and stocking densities were “re‐set” to 10 larvae mL^−1^. Rearing proceeded in these chambers throughout the remaining exposure and oysters were fed daily a mixture of *Tisochrysis lutea* (T‐Iso) and *Chaetoceros neogracile* (Chaet‐B) based on packed cell volumes and age of larvae (Wikfors and Smolowitz 1995). Water changes were conducted every Monday and Friday to maintain water quality while limiting handling stress. During water changes, survival counts were completed volumetrically and larvae subsamples (*N* = 1 mL tank^−1^) were preserved in 95% ethanol for length measurements. Preserved larvae were photographed using an OMAX M82ES Microscope (OMAX Corporation, Kent, WA, USA) equipped with an OMAX 900 digital camera (OMAX Corporation, Kent, WA, USA) and analyzed using the ToupView software (Hangzhou ToupTek Photonics Co. Ltd., Zhejiang, P.R. China). Larval shell measurements were recorded as the furthest point parallel to the hinge. The number of larvae measured from each replicate tank was (*N* larvae replicate tank ^−1^; mean ± standard deviation or SD) 17 ± 6, 70 ± 21, 21 ± 11, and 19 ± 9 at 4, 8, 11, and 15 days postfertilization (dpf), respectively. Recently metamorphosed oysters (juveniles), were measured for the furthest point from the hinge to the ventral margin. Approximately 30 juveniles were measured from each replicate tank, except when mortality limited the number of individuals for sampling (noted in results). Size measurements at 4 and 15 dpf were used to calculate growth rates as μm day^−1^. Experimental conditions were maintained for 22 days at which time ~ 100 pooled juveniles were collected from each replicate and preserved in RNAlater for RNA extraction.

### 2.2 Environmental Conditions

Temperature, salinity, and dissolved oxygen was measured daily for each replicate tank using a handheld Orion Star A329 (Thermo Scientific, Waltham, MA). Seawater samples were collected during water changes in dark polypropylene bottles (250 mL) to measure pH and total alkalinity (TA, μmol kg^−1^) via open‐cell titration (SOP 3b; Dickson et al. 2007). pH was determined colorimetrically at 20°C with m‐cresol purple (Sigma‐Aldrich, St. Louise, M) indicator dye on an UV–VIS spectrophotometer (Cary100, Agilent, Santa Clara, CA, USA) and Andrew Dickson TRIS 37 buffer was used to determine the precision of the pH measurement (± 0.01, *N* = 10). TA was measured using certified HCl titrant (∼0.1 mol kg^−1^, ∼0.6 mol kg^−1^ NaCl; Dickson Lab, Batche 188) on a Metrohm alkalinity titrator (Mettler Toledo) and certified reference material Batch 157 had a precision with ±10 μmol kg^−1^ of the assigned values (*N* = 6). Seawater pH and total alkalinity were input into CO2SYS (Pierrot, Wallace, and Lewis 2011) for the calculation of partial pressure *p*CO_2_ (μatm), and aragonite saturation state (Ω*ar*) using the following constants: K1, K2 from Lueker, Dickson, and Keeling (2000); potassium sulfate from Dickson (1990); and boron from Lee et al. (2010).

### 2.3 Respiration Rates

Approximately 2342 (± 116) larvae from each replicate tank were subsampled without replacement at 24 hpf for respiration rate measurements using Loligo respirometry system (Viborg, Denmark) in 2 mL closed respiration vessels filled with filtered seawater conditioned to each respective treatment. Larvae were distributed to vessels volumetrically, and after being measured, preserved in 95% ethanol to achieve true counts using microscopy (mean ± SD; 2342 ± 116 larvae 2‐mL chamber^−1^). Raw rates of oxygen consumption were estimated using the LoLinR package (Olito et al. 2017), corrected for the mean rate of blank chambers, and converted to appropriate units (ng O_2_ L^−1^ h^−1^). Lastly, rates were normalized to the number of individuals chamber^−1^ (ng O_2_ L^−1^ individual^−1^ h^−1^).

### 2.4 Gene Expression

Whole larvae (24 hpf) and juveniles (22 dpf) were pooled from each replicate tank and fixed in RNAlater (*N* = 24 and 11 samples; respectively). High mortalities under low temperature prevented molecular sampling of juveniles, so only high temperature treatments are represented on day 22. Further, one replicate tank under high temperature × high *p*CO_2_ × high salinity did not have surviving oysters. Whole larvae and juvenile samples were homogenized in Trizol with 0.25 mL 0.5 mm beads by vortexing twice for 25 s. Total RNA was first isolated from pooled tissue homogenate using Trizol reagent (Ambion) before extracted and purified with PureLink RNA Mini Kit and DNAse I (Invitrogen) following manufacturer's instructions. RNA was quantified using the Qubit RNA Broad Range Assay Kit with a fluorometer (ThermoFisher). RNA samples (10 ng μl^−1^) were used for estimation of transcript abundances using TagSeq (Lohman et al. 2016). Library preparation and sequencing of the 35 samples was conducted at the University of Texas Austin, Genomic Sequencing and Analysis Facility on two lanes of Illumina NovaSeq 6000 SR100, targeting a standard coverage of 3–5 million 100 bp single‐end reads per sample. Raw TagSeq reads were trimmed of Illumina adapters, poly‐A sequence, and quality filtered with fastp (Chen 2018); quality control for filter optimization and visual quality control was completed using MultiQC (Ewels et al. 2016). Trimmed reads were mapped to the *C. virginia* genome (NCBI assembly Bioproject PRJNA376014, GCA_002022765.4 C_virginica‐3.0) using HISAT2 (Kim et al. 2015) and resulted in a mapping efficiency of 68%–78%. Transcript abundances were quantified and assembled into a count matrix using Stringtie2 (Pertea et al. 2015).

### 2.5 Gene Expression Analysis

Unique gene (unigene) counts averaged 1,485,793 ± 377,306 reads sample^−1^ (mean ± SD) and 52,002,750 total reads, 10,862 unigenes with at least a single read, or 16.44% of the reference. This master read matrix was split by time point (24 h and 22 days), and each was removed of genes with < 5 counts per million in 50% of samples. These parameters allow pronounced differences in gene expression to remain for analysis (i.e., transcripts absent under low salinity but present under high salinity). Filtering resulted in 1,443,765 ± 341,873 reads sample^−1^ (min‐max; 711,736—2,138,741). There were 4820 and 4936 genes represented in the 24‐h and 22‐day read matrix, ~80% and 76% contained gene name and GO annotation (excluding “uncharacterized” genes).

Weighted Gene Co‐expression Network Analysis (Bioconductor v.3.13 package *WGCNA* v.1.70–3 in R; Langfelder and Horvath 2008, Langfelder and Horvath 2012) was used to identify groups of genes that shared expression at a time point, termed modules. Co‐expression network construction allows an assessment of broad expression‐level directionality and the influence of multiple stimuli (e.g., multifactorial design as salinity × *p*CO_2_ × temperature), as opposed to binary limitations of pairwise differential expression analysis (Zhang and Horvath 2005; Langfelder and Horvath 2008). Using the WGCNA, a signed adjacency matrix was calculated for each read matrix (by time point) using a soft threshold for gene co‐expression network construction (scale‐free fit index *r* ^2^ > 0.9) and minimum module size of 100. Significant module‐treatment associations (< 0.05) were identified via Student's asymptotic p‐value of Pearson correlations (*corPvalueStudent* and *cor* commands from *WGCNA* v.1.70–3 package in R; Langfelder and Horvath 2008, Langfelder and Horvath 2012). Module eigengene‐treatment correlations determined the significant correlation with environmental challenge(s) in this study, and the directionality of expression under different treatment levels. Our goal was to analyze both the main effects and interactions resulting from gene co‐expression modules at each time point, named with colors at each time point as “Day X module color”. If a module eigengene‐treatment correlation is positive, the expression of the module is greater within that treatment. Conversely, if a module eigengene‐treatment correlation is negative, the expression of the module is lower within that treatment. Thus, a significant eigengene‐treatment correlation within a binary treatment framework (i.e., high v. low temperature) presents equal correlation strength in opposing directionalities (i.e., negative under high temperature = positive under low temperature). Student's asymptotic p‐value of < 0.05 for “Module Membership” criteria in WGCNA was used as a data reduction criterion to narrow gene candidates for transcriptome‐to‐phenotype associations. Regularized log transformation (*rlog* command from *DESeq2* v.1.36.0 in R; Love, Huber, and Anders 2014) was used to transform raw transcript counts (discussed below as rlog normalized gene expression) prior to data visualization.

After the data reduction criterion stated above, each co‐expression module that significantly correlated with experimental conditions was assessed for significantly enriched “molecular function” and “biological process” gene ontology (GO) terms (*p* < 0.05) with the Bioconductor package *goseq* in R (v.1.48.0; Young et al. 2010) using Wallenius approximation. The GOslim assignment was used to condense significantly enriched GO terms into hierarchical bins of broader function. A filtering criteria of ≥ 2 genes for each significant “biological process” and “molecular function” GO term was applied before computing GOslim bins. Kyoto Encyclopedia of Genes and Genomes (KEGG; Kanehisa and Goto 2000) was applied to understand higher‐level functional processes of co‐expression modules. Diamond (v.2.0.0; Buchfink et al. 2021) was used to acquire KEGG Orthology (KO) annotation based on sequence relatedness with the *C. virginica* genome query against a Pacific oyster *Crassostrea gigas* protein database Zhang et al. 2012. KEGG terms were assigned based on *C. gigas* hits with highest bit score and lowest e‐value against the *C. virginica* query. These contained a mean bitscore of 781, 70% ± 18% identity, and accounted for 96% of the *C. virginica* genome. Enriched KEGG pathways (adjusted *p*‐value < 0.05) were computed using the Bioconductor package *clusterProfiler* in R (v.4.4.4; Wu et al. 2021) for each co‐expression module significantly correlated with treatment variables. All analysis was completed in R (Posit team 2023). The web interface “*KEGG Mapper*” (Kanehisa and Sato 2020) was used to manually examine enriched pathways.

### 2.6 Statistical Analysis and Data Archive

For carbonate chemistry, we tested for normality with Shapiro Wilk (*p* = 0.05) and equal variance across treatment groups with Levene's Test (*p* = 0.49). A one‐way analysis of variance (ANOVA) was used to test daily averaged Ω*ar* between the eight treatments and a Tukey–Kramer was used for pairwise post hoc comparisons of the significant effect (“*emmeans*” v.1.10.3 in R, Lenth 2019).

Permutational multivariate analysis of variance (PERMANOVA) was used as a nonparametric multivariate approach to analyze physiological data. We used *vegdist* and *adonis2* from the *vegan* R package (v.2.6–4; Oksanen et al. 2022) to estimate euclidean distances of continuous numeric data and run PERMANOVA with 1000 permutations. Models were run separately for each dependent variable (percent survival, respiration rate, and shell length) and time point. Size data was averaged by replicate tank for statistical analysis. Pairwise PERMANOVA models followed significant interaction terms using the same permutational parameters. Data analysis was completed in R (Posit team 2023). Mean live and dead counts between 4 and 15 dpf were converted to binary format to plot the Kaplan–Meier survival estimate (*ggsurvfit* v.1.1.0 R package; Sjoberg et al. 2024). Count data between 4 and 15 dpf (by replicate tank) was binary adjusted and analyzed with Mixed Cox Proportional Hazards Models (*coxme* v.2.2–10 R package; Therneau 2015) for fixed interaction and additive effects of salinity × *p*CO2 × temperature, and in both cases, a single random per‐group intercept of tank replicate. Hazard ratios show the probability of mortality (higher number means more likely to die in that treatment) and were plotted (*survminer* v.0.4.9 R package; Kassambara, Kosinski, and Biecek 2019) as the mean and 95% confidence interval of exponential coefficients output from mixed models.

Analytical code and data files (i.e., physiological data, gene lists and enrichment analysis) are hosted publicly by NOAA National Center for Environmental Information (NCEI Accession 0300468). Raw sequence reads are hosted publicly by the National Center for Biotechnology Information (Accession: PRJNA1207601).

## 3 Results

Ω*ar* differed significantly between the eight treatment groups (*F*_7/40_ = 1017, *p* < 0.001; Table 1). Each group differed, as high “H” versus low “L” salinity × *p*CO_2_ × temperature, with the exception of L × L × H and H × H × L (Table 1 and Figure 1). Statistical results and graphical trends of these data (Figure 1) justified binning treatments categorically based on Ω*ar* and salinity and *p*CO_2_ levels (*N* = 2 treatments bin^−1^) denoted as Ω*ar*‐I, ‐II, ‐III, and ‐IV. Ω*ar* bins represent the following: Ω*ar*‐I = Ω*ar* < 0.5 low salinity × high *p*CO_2_, Ω*ar*‐II = 0.5 < Ω*ar* < 1.0 low salinity × low *p*CO_2_, Ω*ar*‐III = 0.5 < Ω*ar* < 1.0 high salinity × high *p*CO_2_, and Ω*ar*‐IV = Ω*ar* > 1.0 high salinity × low *p*CO_2_. Ω*ar* bins offer descriptive links to significant effects in this study and were employed for eigengene‐treatment correlations in gene network analysis.

**TABLE 1:** Seawater chemistry averaged by each salinity × *p*CO_2_ × temperature treatment.

Experimental Challenge Seawater chemistry										
					Salinity	Temperature	pH	TA	*p*CO_2_	Aragonite
Salinity	*p*CO_2_	Temperature	All	*N*		(°C)	(total scale)	μmol kg SW^−1^	μatm	Saturation
High	Low	High	H × L × H	18	27.56 ± 0.06	27.1 ± 0.1	7.80 ± 0.01	1931.49 ± 7.25	673 ± 6	1.90 ± 0.30^a^
High	Low	Low	H × L × L	18	27.30 ± 0.04	23.1 ± 0.1	7.80 ± 0.01	1943.57 ± 10.81	676 ± 4	1.50 ± 0.020^b^
High	High	High	H × H× H	18	27.56 ± 0.07	27.1 ± 0.1	7.41 ± 0.01	1969.48 ± 8.14	1860 ± 27	0.78 ± 0.01^c^
High	High	Low	H × H × L	18	27.36 ± 0.05	23.1 ± 0.1	7.40 ± 0.01	1977.31 ± 6.79	1850 ± 16	0.66 ± 0.01^d^
Low	Low	High	L × L × H	18	17.40 ± 0.06	27.0 ± 0.1	7.66 ± 0.01	1259.68 ± 5.44	715 ± 7	0.67 ± 0.01^d^
Low	Low	Low	L × L × L	18	17.27 ± 0.05	23.0 ± 0.1	7.65 ± 0.01	1256.78 ± 19.70	732 ± 7	0.55 ± 0.01^e^
Low	High	High	L × H × H	16	17.38 ± 0.07	27.1 ± 0.1	7.28 ± 0.01	1268.84 ± 10.92	1820 ± 36	0.30 ± 0.01^f^
Low	High	Low	L × H × L	14	17.25 ± 0.05	23.0 ± 0.1	7.25 ± 0.01	1258.86 ± 12.79	1870 ± 20	0.23 ± 0.01^g^

*Note:* Salinity and temperature were measured with an Orion Star A329 probes, pH was determined spectrometrically at 20°C, and total alkalinity (TA) by open cell titration. pH and TA were input into CO2SYS to calculate *p*CO_2_ and aragonite saturation (Ω*ar*) at in situ temperature using the following constants: K1, K2 from Lueker, Dickson, and Keeling (2000); potassium sulfate from Dickson (1990); and boron from Lee et al. (2010). Letters represent Tukey's HSD post hoc significant effects (*p* < 0.05) of a one‐way ANOVA.

### 3.1 Physiological Responses

**FIGURE 2:**
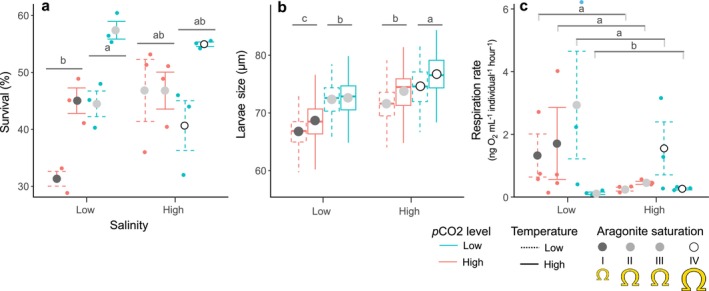
Survival (a), length (b), and respiration rates (c) of oyster larvae at 24 h postfertilization. Survival and respiration rates are shown as mean ± SEM (*N* = 3 tanks treatment^−1^). Larvae size data is shown as boxplots with 25‐75th percentile (boxes) and 1.5x interquartile range of all data (whiskers), mean (circles), and median length (horizontal line). Lower case letters represent pairwise significant differences from PERMANONA interaction terms (*p* < 0.05).

Survival after 24‐h of exposure (*N* = 3 treatment^−1^) was significantly affected by *p*CO_2_ and temperature (*p*CO_2_, *F*_1/16_ = 10.276, *p* = 0.007; temperature, *F*_1/16_ = 22.72, *p* = 0.001) and an interaction of *p*CO_2_ and salinity (*p*CO_2_ × salinity, *F*_1/16_ = 7.528, *p* = 0.022; Table 2). Percent survival relative to within‐treatment counterparts (low versus high *p*CO_2_ and temperature) decreased 14% under high *p*CO_2_ (mean ± SEM; high *p*CO_2_: 42.5% ± 3.7%, low *p*CO_2_: 49.4% ± 4.0%) and 20% under low temperature (low temperature: 40.8% ± 3.4%, high temperature: 51.1% ± 3.0%). Pairwise significant differences between *p*CO_2_ × salinity treatments found survival was 25% lower under high‐*p*CO_2_ × low‐salinity (38.2% ± 6.9%) relative to low‐*p*CO_2_‐low‐salinity (51.0% ± 6.5%; Table 2 and Figure 2). Larval shell length at 24 hpf (*N* = 50 larvae replicate tank^−1^) was significantly affected by *p*CO_2_, temperature, and salinity (*p*CO_2_, *F*_1/16_ = 108.43, *p* = 0.001; temperature, *F*_1/16_ = 18.60, *p* = 0.001; salinity, *F*_1/16_ = 120.43, *p* = 0.001), and an interaction of *p*CO_2_ and salinity (*p*CO_2_ × salinity; *F*_1/16_ = 5.87, *p* = 0.024; Table 2). Larval shell length relative to within‐treatment counterparts were reduced by 5.2% under high *p*CO_2_, 2.2% under low temperature, and 5.5% under low salinity. Pairwise significant differences between *p*CO_2_ × salinity conditions found larvae were ~ 10.5% smaller under high‐*p*CO_2_‐low‐salinity (mean ± SEM; 67.7 ± 0.2 μm) than low‐*p*CO_2_‐high‐salinity (75.7 ± 0.2 μm; Table 2 and Figure 2). Larval shell length at 24‐h was unaffected by temperature. Respiration rates of 24‐h larvae were significantly affected by an interaction between *p*CO_2_ and temperature (*F*_1/16_ = 4.036, *p* = 0.049; Table 2). Pairwise significant differences between *p*CO_2_ × temperature conditions found respiration rates (mean ± SEM) were reduced by 76%–92% under low‐*p*CO_2_‐high‐temperature (0.19 ± 0.04 ng O_2_ L^−1^ individual^−1^ h^−1^; Table 2 and Figure 2).

**TABLE 2:** Mean ± standard error percent survival, size, and respiration rates of oyster larvae from 24 h post fertilization (h) to 15 days post fertilization (d).

Salinity (S)		17				27				
*p*CO_2_ (C)		~1850 μatm		~700 μatm		~1850 μatm		~700 μatm		
Temperature (T)		23°C	27°C	23°C	27°C	23°C	27°C	23°C	27°C	
All		L × H × L	L × H × H	L × L × L	L × L × H	H × H × L	H × H× H	H × L × L	H × L × H	
Time	Measurement									*p*
24 h	Percent survival	31.3 ± 1.3^b^	45.0 ± 2.3^b^	44.5 ± 2.3^a^	57.4 ± 1.6^a^	46.8 ± 5.4^ab^	46.8 ± 3.3^ab^	40.7 ± 4.4^ab^	55.0 ± 0. 4^ab^	C**; T***; C × S*
	Size	66.8 ± 0.3^c^	68.7 ± 0.3^c^	72.4 ± 0.2^b^	72.6 ± 0.3^b^	71.6 ± 0.3^b^	73.8 ± 0.3^b^	74.6 ± 0.3^a^	76.7 ± 0.3^a^	S,C,T**; C × S*
	Respiration rate	1.32 ± 0.69^a^	1.71 ± 1.15^a^	2.93 ± 1.72^a^	0.12 ± 0.04^b^	0.25 ± 0.06^a^	0.45 ± 0.05^a^	1.55 ± 0.85^a^	0.26 ± 0.03^b^	C × T*
4 days	Percent survival	39.0 ± 21.7	36.7 ± 11.9	51.3 ± 13.9	27.7 ± 5.5	58.0 ± 3.1	47.7 ± 4.2	76.7 ± 01.2	63 ± 0.1	S**
	Size	72.5 ± 1.0^bc^	73.2 ± 1.9^c^	80.9 ± 1.0^b^	85.5 ± 1.3^a^	83.2 ± 1.1^bc^	76.1 ± 1.3^c^	83.2 ± 1.0^b^	92.1 ± 1.60^a^	S**; C***; C × T**
8 days	Percent survival	8.0 ± 5.0	6.7 ± 3.3	11.3 ± 2.6	16.0 ± 5.2	34.7 ± 12.3	37.3 ± 5.4	46.7 ± 24.1	32.3 ± 9.0	S**
	Size	89.7 ± 0.7^b^	86.7 ± 0.8^b^	103.6 ± 1.2^ab^	105.1 ± 1.4^a^	102.7 ± 1.0^b^	102.8 ± 1.3^b^	108.1 ± 1.1^ab^	142.2 ± 1.8^a^	S**; C***; C × T*
11 days	Percent survival	5.3 ± 5.0	1.5 ± 0.6	12.1 ± 5.8	10.8 ± 4.0	24.5 ± 7.1	23.1 ± 4.4	30.4 ± 14.7	19.0 ± 4.7	S**
	Size	100.7 ± 1.8^b^	97.2 ± 8.7^b^	118.3 ± 3.3^b^	130.1 ± 5.0^b^	100.5 ± 1.8^b^	144.3 ± 3.6^a^	111.2 ± 2.8^b^	196.9 ± 4.9^a^	S**; T***; S × T**
14 days	Percent survival	0.9 ± 0.9	0.6 ± 0.4	2.7 ± 0.8	8.1 ± 4.4	5.5 ± 3.0	17.7 ± 2.0	17.8 ± 13.1	27.0 ± 6.5	S**
	Size	na	168.3 ± 18.0^b^	130.9 ± 3.3^bc^	231.4 ± 8.5^b^	111.3 ± 1.9^c^	250.6 ± 6.2^a^	133.2 ± 3.2^c^	302.1 ± 5.3^a^	S,T**; S × T*

*Note:* Significant main and interaction PERMANOA effects are shown. Superscript letters represent the results of pairwise PERMANOVA from significant interaction terms.

*p < 0.05; **p < 0.01; ***p < 0.001.

**FIGURE 3:**
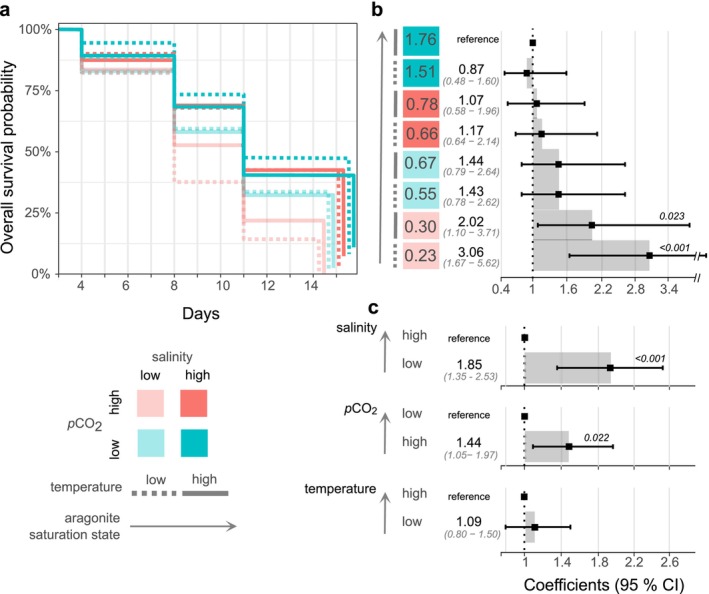
Larval survival from 4 to 15 dpf as Kaplan Meier (a) and Mixed Cox Proportional Hazards models (b and c). The Kaplan Meier plot is staggered visually at 15 dpf to highlight differences in end‐point survival probability between treatments (a). Mixed model coefficients (solid points and text), 95% confidence intervals (grayed parenthesized text), and significant p‐values are shown for the interaction model (b, salinity × *p*CO2 × temperature) and the main effects from the additive model (c, salinity + *p*CO2 + temperature). In all cases, mixed model results are reported relative to the condition with the highest average aragonite saturation state, depicted by directional arrows pointing toward the higher condition (b and c).

**FIGURE 4:**
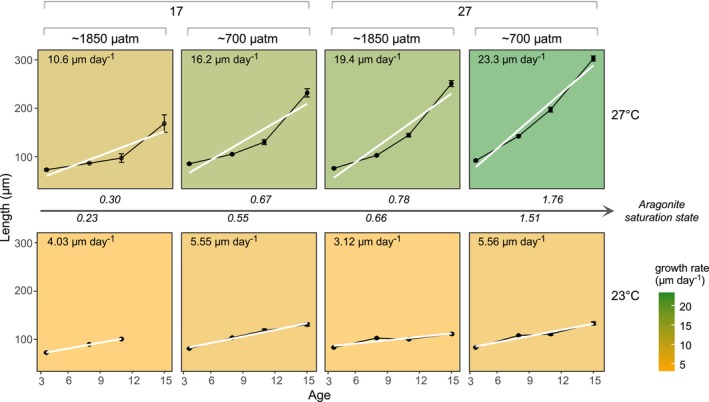
Mean shell length measured from 4 to 15 dpf (μm; mean ± SEM). Plots are faceted by salinity × *p*CO_2_ × temperature treatment, each supplemented visually with linear regressions and background color scale representative of growth rate (μm day^−1^; b) from lowest (yellow) to highest shell growth rate (green). Plots are ordered left to right by low to high average aragonite saturation state supplemented with mean values in *italics*.

Survival from 4 to 15 dpf reduced significantly under low salinity and high *p*CO_2_ and interactively under low salinity × high *p*CO_2_ (Figure 3). There was no main effect of temperature on survival from 4 to 15 dpf (Figure 3). Analyzed by time point, survival on days 4‐, 8‐, 11‐, and 15‐dpf (*N* = 3 replicate tanks treatment^−1^) was affected by salinity with reductions under low salinity by 36%, 71%, 69%, and 64%, respectively (Table 2). Graphical trends for survival to age 15 dpf, when metamorphosis was first observed, demonstrate higher survival with increasing Ω*ar* under low and high temperature with greatest survivorship under the highest aragonite treatments (Ω*ar*‐IV; Figure 4). Kaplan Meier survival analysis and cox proportional hazards showed decreasing survival probability with decreasing Ω*ar* (Figure 3), however at low salinity × high *p*CO_2_ and the lowest Ω*ar* treatment (Ω*ar*‐I) survivorship differed from all other treatments (Figure 3). No larvae survived > 15 dpf under low temperature and Ω*ar*‐I. Data S1 and statistics tables are provided for 24 hpf to 15 dpf (Figure S1 and Tables S1).

Larval shell length at 4 and 8 dpf were significantly affected by *p*CO_2_ and salinity (Day 4: *p*CO_2_, *F*_1/16_ = 32.77, *p* = 0.001; salinity, *F*_1/16_ = 15.06, *p* = 0.001; Day 8: *p*CO_2_, *F*_1/16_ = 19.43, *p* = 0.001; salinity, *F*_1/16_ = 19.10, *p* = 0.002), and an interaction between *p*CO_2_ and temperature (Day 4: *p*CO_2_ × temperature, *F*_1/16_ = 10.08, *p* = 0.006; Day 8: *F*_1/16_ = 5.77, *p* = 0.04; Table 2). Shell lengths at 4 and 8 dpf, respectively, were reduced by 9.5% and 16.2% under high *p*CO_2_ and 5.2% and 14.7% under low salinity relative to within‐treatment counterparts. Pairwise significant differences between *p*CO_2_ × temperature conditions found larvae were 12%–23% smaller under high‐*p*CO_2_‐high‐temperature (Day 4: 74.9 ± 1.1 μm; Day 8: 96.6 ± 0.9 μm) and high‐*p*CO_2_‐low‐temperature (Day 4: 78.2 ± 0.9 μm; Day 8: 97.5 ± 0.7 μm) relative to larvae reared under low‐*p*CO_2_‐high‐temperature (Day 4: 88.3 ± 1.1 μm; Day 8: 124.7 ± 1.4 μm; Table 2). Larval shell lengths at 11 and 15 dpf were significantly affected by temperature and salinity (Day 11: temperature, *F*_1/13_ = 20.20, *p* = 0.001; salinity, *F*_1/13_ = 14.5, *p* = 0.003; Day 15: temperature, *F*_1/10_ = 89.7, *p* = 0.002; salinity, *F*_1/10_ = 16.7, *p* = 0.002) and an their interaction (Day 11: temperature × salinity: *F*_1/13_ = 13.4, *p* = 0.004; Day 15: *F*_1/10_ = 6.8, *p* = 0.025; Table 2). Shell lengths at 11 and 15 dpf were reduced by 33% and 51% under low temperature and 18% and 14% under low salinity relative to within‐treatment counterparts. Pairwise significant differences between temperature × salinity conditions found larvae were largest under high‐temperature‐high‐salinity (Day 11: 170.9 ± 3.6 μm; Day 15: 270.2 ± 4.8 μm) and were 24%–38% and 17%–54% smaller under all other treatments (Table 2). Shell growth rates between 4 and 15 days dpf were < 6 μm day^−1^ under low temperature and increased from 11 to 23 μm day^−1^ under Ω*ar*‐I to Ω*ar*‐IV under high temperature (Figure 4). Data S1 and statistics tables are provided for 24 hpf to 15 dpf (Figure S2 and Table S2). Shell length at age 19 and 20 dpf are reported for high temperature treatments (Figure S4), but no larvae survived > 15 dpf under low temperature and Ω*ar*‐I.

### 3.2 Co‐Expression Network Analysis Overview

Data reduction of gene network modules based on the Student's asymptotic *p*‐value < 0.05 for module membership contained Pearson's correlation coefficient for module membership (mean ± SEM) of 0.64 0.02 (*N* = 6 modules) for 24‐h larvae and 0.80 ± 0.01 (*N* = 6 modules) for the 22‐day juveniles; data reduction resulted in ~55%–60% of genes removed from network modules to focus on more conservative eigengene‐treatment relationships. Functional enrichment and pathway analysis are reported for reduced gene sets in network modules correlated with main effects and categorical Ω*ar* bins.

### 3.3 24‐h Oyster Larvae

Network analysis of gene expression in 24‐h oyster larvae yielded six significant co‐expression modules (Figure S3). Of these six modules, three were significantly correlated with the main effects of salinity and Ω*ar* bins (Day 1 modules brown, blue, and turquoise) of which modules blue and turquoise were also correlated with temperature (Figure S3). Review Appendix 1 for a list of genes and gene families included, but not limited to, those within enriched KEGG pathways and GO terms for the following modules.

**FIGURE 5:**
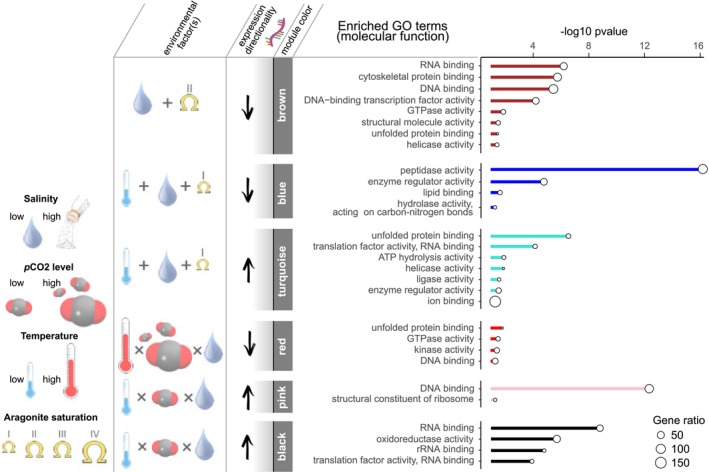
Significantly enriched molecular function GO terms for co‐expression modules, groups of genes that shared expression pattern, of 24‐h oyster larvae. Significant module‐treatment correlations are shown alongside the directionality of expression and cases of multiple main‐treatment correlations are depicted as “+”. For modules absent of main‐treatment correlations, an interaction term is given as “×”. Roman numerals for aragonite saturation state represent the four major treatment bins (as shown in Figure 1).

Day 1 brown, representing low gene expression under low salinity and Ω*ar*‐II (Figure S3), was enriched for pathways “spliceosome” (*N* = 16 genes) and “motor proteins” (*N* = 14; Table S4). GO analysis included significant enrichment in the following terms: cellular nitrogen compound metabolic process, RNA/DNA binding proteins, transcription factor activity, mRNA processing, GTPase activity, cytoskeletal and structural molecule binding, unfolded protein binding, and helicase activity (Figure 5).

Day 1 module blue, representing low gene expression under low salinity, low temperature, and Ω*ar*‐I (Figure S3), was enriched for pathways “glutathione metabolism” (*N* = 7), “lysosome” (*N* = 17), “phagosome (*N* = 10)”, and “oxidative phosphorylation” (*N* = 5; Table S4). GO analysis of Day 1 module blue included significant enrichment in the following terms: metabolic processes (cellular nitrogen compound, lipid, and carbohydrate), catalytic activity (hydrolase and peptidase activity), enzyme regulator activity, iron transport/binding, homeostatic process, oxidoreductase activity, and generation of precursor metabolites and energy (Figure 5).

Lastly, Day 1 module turquoise, representing higher expression under low salinity, low temperature, and Ω*ar*‐I (opposite pattern of Day 1 module blue; Figure S3), was enriched for pathways “protein export” (*N* = 4), “spliceosome” (*N* = 8), “phosphatidylinositol signaling system” (*N* = 7, Table S4). GO analysis included significant enrichment in the following terms: protein folding, enzyme regulator activity, cellular component assembly, unfolded protein binding, ion binding, and cellular nitrogen and sulfur compound metabolic process (Figure 5).

### 3.4 22‐Dpf Juvenile Oysters

Network analysis of expression in 22‐day juvenile oyster yielded six significant co‐expression modules (Day 22 modules blue, red, salmon, tan, green, and turquoise; Figure S4), each significantly correlated with Ω*ar* bins. Review Appendix 1 for a list of genes and gene families included, but not limited to, those within enriched KEGG pathways and GO terms for the following modules.

**FIGURE 6:**
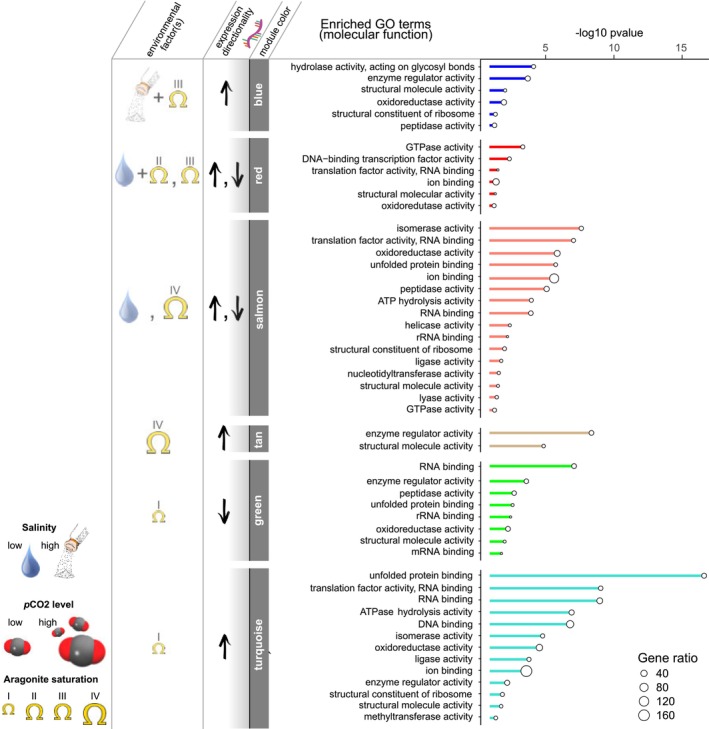
Significantly enriched molecular function GO terms for co‐expression modules, groups of genes that shared expression pattern, of 22‐day juveniles. Significant module‐treatment correlations are shown alongside the directionality of expression. Cases of multiple main‐treatment correlations are depicted as “+”. Roman numerals with aragonite saturation state represent the four major treatment bins (as shown in Figure 1). Only high temperature is represented at this timepoint, as no larvae metamorphosed under low temperature.

Day 22 module tan, representing genes highly expressed under Ω*ar*‐IV (Figure S4), was enriched for pathways “folate biosynthesis” (*N* = 2), “ATP‐dependent chromatin remodeling” (*N* = 4), and “motor proteins” (*N* = 5; Table S4) and GO terms enzyme regulator activity and structural molecule activity (Figure 6). Day 22 module turquoise, representing genes highly expressed under Ω*ar*‐I (Figure S4), was enriched for pathways “citrate cycle (TCA cycle)” (*N* = 9), “proteasome” (*N* = 10), “spliceosome” (*N* = 27), “oxidative phosphorylation” (*N* = 16), “ATP‐dependent chromatin remodeling” (*N* = 14), and “carbon metabolism” (*N* = 15; Table S4). Enriched GO terms and GOslim categories in Day 22 module turquoise involved: protein targeting and modification, cellular nitrogen compound and cellular amino acid metabolic process, generation of precursor metabolites and energy, unfolded protein and ion binding, and oxidoreductase and methyltransferase activity (Figure 6). Day 22 module green, representing lower expression under Ω*ar*‐I (Figure S4), was enriched for pathways “ribosome” (*N* = 39) and “oxidative phosphorylation” (*N* = 7) and the following GO terms and GOslim categories: cellular nitrogen compound metabolic processes, hydrolase activity, molecular function regulator, RNA binding (mRNA and translation factor activity) (Figure 6). A single KEGG pathway was enriched (“ribosome” *N* = 39) attributed to ribosomal proteins (Table S4).

Day 22 modules blue, red, and salmon were additionally correlated with the main effect of salinity, representative of divergent gene expression patterns under multiple challenges. (Figure 6). Day 22 module blue, representing genes highly expressed under Ω*ar*‐III and high salinity (low expression under low salinity; Figure S4), was significantly enriched in the following GO terms and goSlim categories: enzyme regulator, hydrolase, structural molecular, oxidoreductase, and peptidase activity, carbohydrate and lipid metabolic processes, and generation of precursor metabolites and energy (Figure 6).

Day 22 module red, representative genes highly expressed under low salinity and Ω*ar*‐II and reduced expression under Ω*ar*‐III (Figure S4), was enriched for pathways “protein export” (*N* = 4) and “endocytosis” (*N* = 11; Table S4) and included significant enrichment in the following GO terms and GOslim categories: ion binding, cellular nitrogen compound metabolic process, transport, GTPase activity, and oxidoreductase activity (Figure 6).

Day 22 module salmon, representing genes abundant under low salinity and suppressed under Ω*ar*‐IV (Figure S4), was enriched for pathways “proteasome” (*N* = 15), “mRNA surveillance pathway” (*N* = 10), and “oxidative phosphorylation” (*N* = 9; Table S4). GO analysis of Day 22 module salmon, included but not limited to, the following enriched terms: protein folding, small molecular metabolic process, cellular nitrogen and sulfur compound binding, homeostatic process, transport, oxidoreductase and peptidase activity, and ion binding (Figure 6).

## 4 Discussion

By isolating the physical factors of salinity, temperature, and *p*CO_2_, this study helps to disentangle the compounded effects seen with changing environmental conditions. Using Ω*ar* as a descriptive factor to explore phenome‐to‐transcriptome linkages highlights how oyster larvae respond to multifactorial environmental challenges, a more ecologically realistic climate change scenario. Factors that reduced Ω*ar* in this study, low salinity, elevated *p*CO_2_, and low temperature (Figure 1), each slowed early development and reduced survival in the eastern oyster *C. virginica* . Thus, larval‐to‐juvenile performance (shell growth and percent survival to metamorphosis) was dependent on Ω*ar* with near‐linear physiological implications as Ω*ar* undersaturation intensified (Figures 3 and 4). Although the negative effects of undersaturated Ω*ar* are well documented for bivalve larvae (Waldbusser et al. 2015a), how genes are expressed under coupled environmental stimuli and in the context of Ω*ar* are less understood and were a primary focus of this study.

### 4.1 Organismal Response to Low Ωar

Ω*ar* undersaturation < 1.0 (Ω*ar*‐I, Ω*ar*‐II, and Ω*ar*‐III, Figure 1) negatively affected growth and survivorship during larval development. The full factorial design of this study provided an opportunity to evaluate the effects of coastal freshening and coastal acidification together and in isolation. When Ω*ar* was reduced only moderately (Ω*ar*‐II and Ω*ar*‐III), we observed lower survival to settlement and slowed shell development under hyposaline (low *p*CO_2_ × low salinity; Ω*ar*‐II) relative to acidification (high *p*CO_2_ × high salinity; Ω*ar*‐III). This suggests greater energetic costs under low salinity. Postembryonic and presettlement stages (24 hpf and 15 dpf) were affected by *p*CO_2_ × salinity and the full experimental interaction (*p*CO_2_ × salinity × temperature), with lowered growth and survival when Ω*ar* < 1.0. Thus, conditions favorable for survival and shell growth were defined by main effects (i.e., high salinity) or interaction terms (i.e., low *p*CO_2_ × high salinity) when Ω*ar* was above 1.0 (Ω*ar*‐IV, Figure 1). Similar studies employing low and high salinity, temperature, and *p*CO_2_ conditions found delayed development and more abnormalities in larval Pacific oysters *Crassostrea gigas* (Ko et al. 2014) and brooding flat oysters *Ostrea angasi* (Cole et al. 2016) when Ω*ar* was lowered. Importantly, an increase in temperature + 4°C–6°C in these studies exacerbated effects of elevated *p*CO_2_ and low salinity (i.e., 24°C and 30°C in Ko et al. 2014; 26°C and 30°C in Cole et al. 2016); larval *C. virginica* also grow more slowly at 30°C than at 25°C (McFarland et al. 2022b). In contrast, *C. virginica* larvae grew faster and metamorphosed successfully at the chosen high temperature level of 27°C. Faster larvae‐to‐juvenile development under approximate fertilization temperature (~28°C) relative to our chosen low temperature condition (23°C) suggests a possible cold shock from thermal mismatch between the pre‐ and postfertilization environments and is further discussed in our transcriptomics results. Altogether, the effects of low salinity, elevated *p*CO_2_, and low temperature highlight Ω*ar* as a unifying metric for multifactorial challenges, especially for marine calcifiers.

### 4.2 Transcriptional Response to Low Temperature

Genes affected by temperature in 24‐h larvae can provide valuable insights, as the low temperature treatment yielded 100% mortality by age 22 days, preventing transcriptomic sampling at 22 dpf. Transcriptional patterns at 24 hpf were responsive to temperature and salinity. Gene sets affected by temperature were compared to salinity variation at constant temperature to discern unique genes affected by temperature alone. Larvae 24‐h hpf suppressed expression of fructose bisphosphate aldolase and malate dehydrogenase and increased abundance of carnosine synthase‐1 (*CRNS‐1*) under low temperature. *CRNS‐1* functions in free amino acid metabolism of *B*‐alanine. In the mussel *Mytilus coruscus* high levels of *B*‐alanine are necessary to enhance carbohydrate metabolism and meet energy demands (Wang et al. 2021). Expression of *CRNS*‐1 under low temperature is interpreted as a regulatory response in selected lines of the pearl oyster *Pinctada fucata martensii* (Wang et al. 2018) but was accompanied in this study by suppressed carbohydrate metabolism in *C. virginica* at low temperature. Expression of *CRNS*‐1 over carbohydrate metabolism may infer a maladaptive metabolic shift from glycolysis and poses important considerations for predictive molecular candidates, as low temperature elicited a severe delay in larval growth and complete mortality at the juvenile stage (22 dpf). Lastly, low temperature stimulated expression of dnaJ homolog, a protein chaperone, and glutathione components involved in oxidative stress response. Cold stress can elevate transcription of molecular chaperones in the Pacific oyster *C. gigas* (Zhu et al. 2016) and antioxidants in the noble scallop *Chlamys nobilis* (Tan et al. 2019). Further, the pearl oyster *P. martensii* changes expression of molecular chaperones in response to temperature variation (Wang et al. 2019). In summary, the functional transcriptome at 24 hpf may incur regulatory and metabolic costs suggesting that exposure outside of fertilization temperature could limit energy for larval growth and development.

### 4.3 Transcriptional Response to Reduced Ωar

Gene expression analysis of larval and early juvenile stages reared at high temperature provided new insights into phenome‐to‐transcriptome responses under reduced salinity and acidified seawater. Transcriptomics data reinforced organismal responses and elucidated factor‐dependent gene candidates in response to these Ω*ar*‐lowering factors, both combined and in isolation. There were two expression patterns in this study: (1) bidirectional response to severe undersaturation (Ω*ar*‐I) and (2) bidirectional response moderate undersaturation (Ω*ar*‐II and Ω*ar*‐III), meaning unique gene sets that were abundant or suppressed.

**FIGURE 7:**
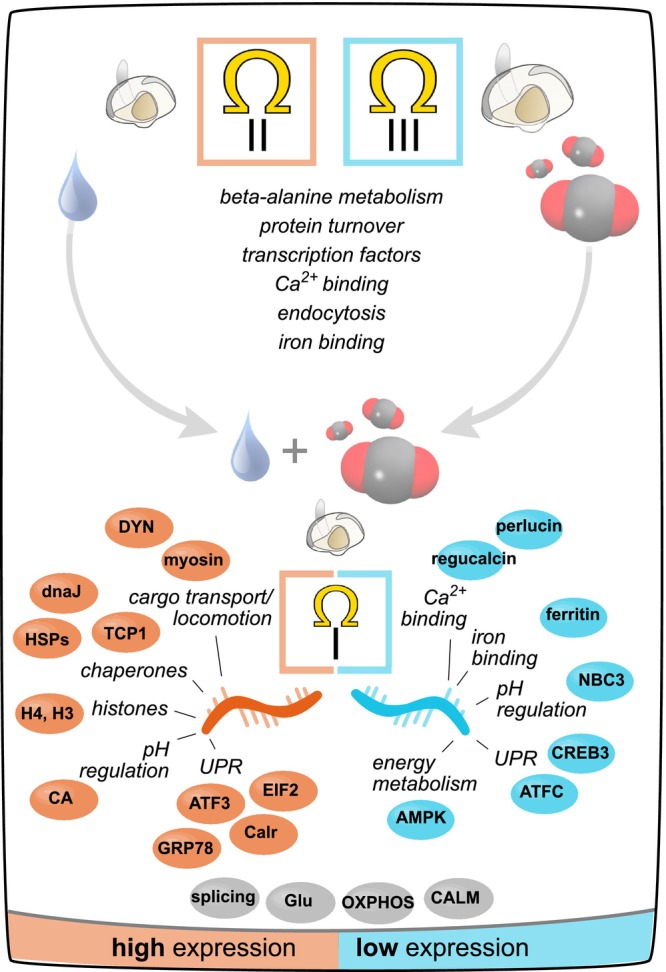
Summary of enriched pathways and transcripts that were expressed (orange), suppressed (blue), or bi‐directional (gray) for different Ω*ar* bins. Three of the four Ω*ar* bins (low to high as roman numerals I‐III) represent severely undersaturated driven by high *p*CO_2_ and low salinity (Ω*ar*‐I), moderately undersaturated driven by low salinity (Ω*ar*‐II), and moderately undersaturated driven largely by high *p*CO_2_ (Ω*ar*‐III). Categorical bins for Ω*ar* are displayed with the direction of gene expression (orange and/or blue box), cartoons for high v. low salinity and/or *p*CO_2_ conditions, and relative oyster shell size. Abbreviations are as follows in alphabetical order: 5′‐AMP‐activated protein kinase “AMPK”, activated transcription factor 3 and of chaperones “ATF3” and “ATFC”, carbonic anhydrase “CA”, calmodium “CALM”, calreticulin “Calr”, cyclic AMP‐responsive element‐binding protein 3 “CREB3”, dnaJ homologs “dnaJ”, dynein proteins “DYN”, eukaryotic translation initiation factor 2 “EIF2”, 78 kDa glucose regulated protein “GRP78”, glutathione proteins “Glu”, histones 3 and 4 “H3” and “H4”, heat shock proteins “HSPs”, sodium‐bicarbonate cotransporter 3 “NBC3”, genes involved in oxidative phosphorylation “OXPHOS”, and T‐complex protein 1 “TCP1”.

Elevated gene expression at Ω*ar*‐I involved genes responsible for microtubule transport and ciliary locomotion, protein folding, histone modifications, and acid–base balance (Figure 7). First, dynein and kinesin family proteins and protein chaperones were abundant in both larvae and early juveniles under Ω*ar*‐I. Dynein and kinesin family proteins are cytoplasmic motor proteins that function in intracellular transport, ciliary beating, and maintenance of cellular structure (reviewed in Vale 2003). Protein chaperones, such as T‐complex proteins (TCPs) and heat‐shock proteins (e.g., 90, 75, and 10 kDa), repair misfolded proteins and serve as an indicator of disrupted proteostasis. Similar to these data on *C. virginica* , clams were shown to upregulate dynein and TCP1 under hypoxia and heat stress (e.g., as in *Mercenaria mercenaria* and *Panopea globosa*; Hu et al. 2022; Juárez et al. 2018). Axonemal dynein family proteins are suppressed under low salinity in the blue mussel *Mytilus edulis* (Campos et al. 2016) and under elevated *p*CO_2_ in the Sydney rock oyster *Saccostrea glomerata* (> 1000 μatm *p*CO_2_; Goncalves et al. 2017a; Goncalves, Thompson, and Raftos 2017b). A low abundance of dynein motor proteins may reduce water circulation driven by ciliary beating (Campos et al. 2016), linked to known effects of moderate hyposalinity on metabolic depression and bradycardia (i.e., in *M. edulis* ; Stickle and Sabourin 1979; Bakhmet, Berger, and Khalaman 2005). Suppression of ciliary beating is also linked to decreased particle clearance rates and fitness in the blue mussel *Mytilus edulis* under OA (Ω*calcite* 0.5 and > 200 μatm *p*CO_2_; Meseck et al. 2020). In contrast to our findings, elevated *p*CO_2_ exposure reduced expression of T‐complex protein 1 in the Sydney rock oyster (856 μatm *p*CO_2_; Thompson et al. 2016), suggesting lower capacity for protein homeostasis. Altogether, a higher transcript abundance of microtubule transport proteins and chaperones by *C. virginica* larvae could be attributed to active aerobic performance and protection from protein degradation during acidification under reduced salinity (and thus low Ω*ar*). Ciliary beating suppression may be critical for mollusks under OA (Meseck et al. 2020), thus future research on dynein and kinesin is needed to elucidate their roles in adaptive stress response.

Histone and histone‐lysine modifiers were also abundant under Ω*ar*‐I (Figure 7). Histone availability bolsters immunity in *C. virginica* (i.e., H2B; Seo et al. 2010); whereas, a deficit in histone supply can deteriorate chromatin structures and affect cell fitness in model taxa (Prado, Jimeno‐González, and Reyes 2017). The role of histones in chromatin remodeling, and thus gene regulation and epigenetics, emphasizes their putative importance under environmental change. For example, juvenile Pacific geoduck clams *Panopea generosa* primed to elevated *p*CO_2_ increased shell growth and transcribed histone‐lysine modifiers (Gurr et al. 2022). Moreover, histone modifiers were also elevated during the long‐term hypoxia challenge in the pearl oyster *Pinctada fucata martensii* (Yang et al. 2023). Lastly, oyster juveniles under Ω*ar*‐I increased expression of carbonic anhydrase, an acid–base regulatory protein. Specific to marine calcifiers, carbonic anhydrase serves as a biomarker of global change as a consequence of its importance for biocalcification, CO_2_ sensing/excretion, and ion regulation (reviewed in Zebral et al. 2019). Transcription of carbonic anhydrase by the Pacific oyster *C. gigas* does not translate to increased enzymatic activity under high *p*CO_2_ alone (Wang et al. 2017), whereas coupled environmental challenges can excite activity (Ivanina et al. 2013). Thus, a high transcript abundance at Ω*ar*‐I (high *p*CO_2_ × low salinity) herein suggests both osmoregulatory and acid–base responses are plausible. Lastly, shell matrix proteins were uniquely abundant at 24 hpf (protein pif‐life) and 22 dpf (NA^+^ K^+^ ATPases, chitinase, and calumenin). *C. gigas* similarly demonstrate timing‐by‐environment demand for transcription of shell matrix proteins during Ω*ar* undersaturation (0–3 v. 15–24 hpf at Ω*ar* 0.59; Wright‐LaGreca, Mackenzie, and Green 2022). Together, a continuous transcriptional demand for ciliary locomotion/transport, proteostasis, and acid–base and transcriptional regulation may allow oyster larvae and early juveniles to mediate effects of aragonite undersaturation.

Gene suppression at Ω*ar*‐I was attributed to calcium binding, iron metabolism, immune response, and signal transduction (Figure 7). Calcium‐binding proteins associated with the acquisition, transport, and assimilation of calcium (e.g., calmodulin and sodium/calcium exchangers), essential for chitin production of the shell matrix (Weiss et al. 2000; Yan et al. 2007) and shell deposition during oyster larval development (Ramesh et al. 2019). Perlucin and regucalcin were consistently suppressed by larvae and juveniles in Ω*ar*‐I. Perlucin promotes precipitation of CaCO_3_ crystals in nacreous microstructures (Suzuki and Nagasawa 2013; Weiss et al. 2000) contributing to shell biosynthesis in marine calcifiers (Hofmann, O'Donnell, and Todgham 2008); it is expressed in epithelial cells of bivalve mantle tissue (Lin et al. 2013; Song et al. 2022). Resilience of the clam *Panopea globosa* to *p*CO_2_‐induced Ω*ar* undersaturation (~2492 μatm and Ω*ar* ~ 0.57) is attributed to compensatory expression of perlucin to slow shell dissolution/damage (López‐Landavery et al. 2021). Regucalcin, a calcium‐binding protein, is also expressed within mantle tissues in bivalves (Shi et al. 2013); its functions include maintenance of intracellular Ca^2+^ homeostasis (Marques et al. 2014) and prevention of apoptosis (Lian et al. 2020). Increased expression of regucalcin under heat challenge (Yang et al. 2017) suggests improved cytoprotective activity; however, reduced expression can occur under elevated *p*CO_2_ (~2000 μatm; Wei et al. 2015). Lastly, low transcript abundance of sodium‐bicarbonate cotransporter 3 (NBC3) by both larvae and juveniles suggests that exposure to low Ω*ar* may impair membrane transport of bicarbonate in epithelial cells. Interpreted jointly, suppression of calcium‐binding and pH‐regulatory proteins under low Ω*ar* may signify compromised shell biomineralization and pH homeostasis; concurrent with slower growth rates. Our findings reinforce the functional association of perlucin and regucalcin with shell precipitation/repair and emphasize gene candidates linked to early OA sensitivity.

Iron binding proteins were also suppressed by both larvae and juveniles in Ω*ar*‐I. Ferritin‐related proteins (soma ferritin, ferritin low subunit, etc.) are integral to iron homeostasis and enable storage of soluble iron (ferrous Fe^2+^), thus making iron insoluble or less bioavailable (ferric Fe^+3^; Andrews et al. 1992). The Fenton reaction is dependent upon excess soluble iron (Stohs and Bagchi 1995) and produces carbonate‐anion‐radicals in the presence of bicarbonate (Illés et al. 2019). This reaction guides new insights on the nature of intracellular free radicals linked to biological systems (Vijay, Sharma, and Meyerstein 2023) and is postulated as a source of intracellular oxidative damage under OA (Tomanek et al. 2011). Thus, a high abundance of ferritin‐like storage proteins may prevent excess‐iron induced toxicity (via the Fenton reaction) and oxidative damage under environmental challenge. For example, exposure to elevated *p*CO_2_ elicits ferritin induction in the white shrimp *Litopenaeus vannamei* (Zhou et al. 2008) and elevated transcript abundance in larval *C. virginica* (Barbosa et al. 2022) and adult *S. glomerata* (Goncalves, Thompson, and Raftos 2017b). In contrast, low transcription of iron‐binding proteins under low Ω*ar* suggests that the benefits of readily‐available, soluble iron (for metabolism) may outweigh the costs of associated free‐radical risk in *C. virginica—*or antioxidants such as glutathione were expressed at sufficient levels to ameliorate oxidative stress (Figure 7).

Ω*ar*‐I also elicited both persistent abundance and suppression of transcripts involved in the unfolded protein response (UPR; Figure 7). The UPR is a cascade of signaling molecules, transcription factors, and stress‐response genes induced by endoplasmic reticulum (ER) stress. Genes with functions in quality control for newly produced proteins (calreticulin), mitigation of misfolded protein aggregates (78 kDa glucose‐regulated protein), and response following ER stress (eukaryotic translation initiation factor 2 alpha, EIF2‐*a*, and activating transcription factor 3, ATF3) were abundant under low Ω*ar*. Broadly, EIF2‐*a* signals for the downstream translation of ameliorative proteins (Malhotra and Kaufman 2007) assisted by the transcription factor ATF‐3 (Jiang et al. 2004), suggesting that oyster larvae may activate UPR under low Ω*ar* exposure. In contrast, cyclic AMP‐responsive element binding protein 3 (CREB3) was suppressed under low Ωar; it is implicated in cellular homeostasis (Smith et al. 2023). Evidence remains equivocal regarding how UPR applies to mollusks under environmental challenges, as many UPR‐related genes are upregulated under acute thermal challenge (i.e., in *C. gigas* ; Kawabe et al. 2010; Zhang et al. 2012; Yang et al. 2017; reviewed in Huang et al. 2018) yet can be suppressed under elevated *p*CO_2_ (i.e., *S. glomerata* ; Thompson et al. 2016). Interpreted jointly, either ER stress and/or an imbalance of signaling molecules (Ca^2+^, ROS, and ATP) under low Ω*ar* exposures appeared to incur transcriptional costs in the activation of the UPR.

Importantly, the design of this study can disentangle effects of salinity and acidification challenges in Ω*ar*‐II and Ω*ar‐*III (Figure 7). Lower survival to settlement and slowed shell development under hyposaline (low *p*CO_2_ × low salinity; Ω*ar*‐II) relative to acidification (high *p*CO_2_ × high salinity; Ω*ar‐* III) suggests greater calcification costs under low salinity. Independent of the abiotic factor, however, oyster larvae and early juveniles may also be sensitive to subtle differences in carbonate dissolution as low salinity‐induced undersaturation, or Ω*ar*‐II, reduced Ω*ar* by −0.1 relative to elevated *p*CO_2_ in Ω*ar‐*III (Figure 1 and Table 1). As the second bidirectional pattern in this study, gene expression in Ω*ar*‐ II *and* Ω*ar‐* III elucidated transcripts affected by hyposaline (low *p*CO_2_ × low salinity; Ω*ar*‐II) and acidification in isolation (elevated *p*CO_2_ × high salinity; Ω*ar*‐III). Hyposaline‐induced moderate undersaturation, as Ω*ar*‐II, was associated with expression of *B*‐alanine metabolism, endocytosis, calcium binding, and iron metabolism (Figure 7). *CRNS‐1*, involved in small amino acid metabolism, was also abundant under Ω*ar*‐I (high *p*CO_2_ × low salinity) suggesting a response to low salinity independent of *p*CO_2_ or Ω*ar* level. Bivalves possess limited osmoregulatory capacity by regulating the accumulation or depletion of free amino acids to adjust osmotic pressure (Bishop, Greenwalt, and Burcham 1981). In brief, mollusks increase osmolarity of intracellular space (cytosol) by accumulating free amino acids (i.e., proteolysis); whereas, free amino acids are excreted or metabolized under low salinity to reduce osmolarity (Hawkins and Hilbish 1992; Pourmozaffar et al. 2020). Alanine is a small amino acid that acts as an osmotic solute to regulate cell volume (Pierce and Amende 1981). For example, hypo‐ and hypersaline adaptations of *Crassostrea gigas* involve synchronous fluctuations of free amino acids, primarily a reduction of taurine and an increase in alanine, respectively (Hosoi et al. 2003). Thus, high gene expression of *CRNS‐1* under low salinity infers a demand to reduce osmolarity through *B*‐alanine metabolism; thereby reinforcing alanine accumulation as a putative adaptation to high salinity in oysters (Pierce, Rowland‐Faux, and O'Brien 1992). Similarly, osmoconformers such as the *C. gigas* and the Mediterranean mussel *Mytilus galloprovincialis* increase free amino acid metabolism under low salinity stress (Icagasioglu et al. 2022; Meng et al. 2013), similar to our findings. Hyposaline conditions were also linked uniquely to expression of charged multivesicular subunits that form the ESCRT‐III complex, a group of proteins involved in sorting, multivesicular body formation, and eventual transport of proteins for lysosomal degradation (Fader and Colombo 2009). Together, moderate Ω*ar* under low salinity (Ω*ar*‐II) allowed for +6 um shell growth day^−1^ and + 8% survival to set compared to Ω*ar*‐I (Figure 4), and was attributed, at least in part, to adaptations in biomineralization, proteostasis, and osmoregulation under salinity challenge. Lastly, soma ferritin was suppressed under elevated *p*CO_2_‐induced moderate Ω*ar* (Ω*ar*‐III) and Ω*ar*‐I (Figure 7) suggesting that reduced expression was affected by elevated *p*CO_2_ independent of salinity or Ω*ar* level. Whether iron binding and the Fenton reaction interact to affect organismal fitness during OA encounters remains unclear.

## 5 Conclusion

We exposed *C. virginica* from embryo to early juvenile stage under low and high salinity × *p*CO_2_ × temperature to generate an Ω*ar* spectrum and elucidate transcriptional underpinnings of phenotypic response. A − 4°C decline in temperature suppressed expression of glucose metabolism in oyster larvae precursory to full mortality by early juvenile stage, suggesting a subtle temperature shift, independent of seawater salinity and Ω*ar*, can compromise energy metabolism and recruitment success. Physiological fitness (shell growth rate and survival to juvenile stage) decreased with Ω*ar* suggesting that unique responses are elicited by major Ω*ar*‐reducing factors: low salinity, elevated *p*CO_2_, and low salinity × elevated *p*CO_2_. Conditions that severely affected Ω*ar* (low salinity × elevated *p*CO_2_; Ω*ar*‐I) elicited unique transcriptional patterns that putatively incurred costs for fitness; these included suppression in bicarbonate transport and biomineralization of calcareous microstructures and abundance in ciliary locomotion, proteostasis, and histone modifiers. Ω*ar* “reducers” in isolation (Ω*ar* < 1 low salinity or elevated *p*CO_2_; Ω*ar*‐II and Ω*ar*‐III) induced transcriptional demand for osmoregulation and endocytosis under hyposaline conditions whereas iron binding was reduced under acidification. Although fitness was affected by Ω*ar* undersaturation, these transcriptional patterns in survived juvenile represent putative functions that confer tolerance to environmental challenge. As oysters emerge in the spotlight for food security and habitat health, sensitive ‐omic features are integral for the development of climate‐ready strains and selective breeding programs. Our findings offer novel insights into gene candidates affecting growth and recruitment success within systems that experience seasonal warming, freshwater effluent, and coastal acidification.

## Author Contributions

**Samuel J. Gurr:** data curation (lead), formal analysis (lead), methodology (equal), writing – original draft (lead), writing – review and editing (lead). **Shannon L. Meseck:** conceptualization (lead), data curation (lead), formal analysis (supporting), funding acquisition (lead), investigation (equal), methodology (equal), project administration (lead), writing – review and editing (equal). **Genevieve Bernatchez:** data curation (equal), investigation (equal), methodology (equal), writing – review and editing (supporting). **Dylan Redman:** data curation (equal), methodology (equal). **Mark S. Dixon:** data curation (equal), methodology (equal). **Lisa Guy:** data curation (equal), methodology (equal). **Aaron MacDonald:** investigation (equal). **Sheila Stiles:** conceptualization (lead), investigation (equal), methodology (equal), project administration (lead), writing – review and editing (supporting). **Katherine McFarland:** conceptualization (lead), data curation (lead), formal analysis (supporting), funding acquisition (lead), investigation (equal), methodology (equal), project administration (lead), writing – original draft (supporting), writing – review and editing (lead).

## Conflicts of Interest

The authors declare no conflicts of interest.

## Supporting information


Data S1.

## Data Availability

Raw sequence reads are publicly available hosted by the National Center for Biotechnology Information (Accession: PRJNA1207601; BioProject: Transcriptome‐to‐phenome response of larval Eastern oysters under multiple drivers of aragonite undersaturation). All other data are publicly available hosted by the National Oceanic and Atmospheric and Administration National Center for Environmental Information (NCEI Accession 0300468).

## Appendix 1

### 24‐h Oyster Larvae

Each WGCNA module significantly correlated with experimental treatments was assessed for molecular functions and biological processes of gene groups. Genes associated with enriched pathways and GO terms included, but not limited to, the following proteins, protein families, and functions (parsed by module “color”):

#### Brown

Transcriptional modifiers (transcription factor GATA, homeobox proteins, forkhead box protein K1, T‐box proteins, and histone acetyltransferases), microtubule trafficking and locomotion (myosin heavy chain, paramyosin, unconventional myosin‐VI, kinesin, dynein heavy chain [5 and 10], and dynein beta chain proteins [ciliary and flagellar outer arm]), Ca^2+^‐dependent cellular processes (calcium‐transporting ATPase, sodium/calcium exchanger 3, and calcium homeostasis endoplasmic reticulum), protein chaperones (dnaJ homolog, cold‐sock domain‐containing protein, stress‐70 mitochondrial), unfolded protein response (UPR; cyclic AMP‐responsive element‐binding protein 3), cellular metabolism (5′‐AMP‐activated protein kinase), E3 ubiquitin ligases (TRIP12, RBBP6, HUWE1), splicing factors, and actin binding and housekeeping proteins (advillin, titan and regulator of nonsense transcripts).

#### Blue

Carbohydrate metabolism/glycolysis (fructose bisulfate aldolase, malate dehydrogenase), oxidative phosphorylation (ATP synthases, V‐type proton ATPase subunit, NADH dehydrogenase, succinate dehydrogenase, cytochrome c oxidase), pH regulation (sodium‐bicarbonate transporter 3), regulation of oxidative stress (glutathione peroxidase 1 and 2, metallothionein, glutathione S‐transferase, peroxiredoxin, S‐crystallin SL11, isocitrate dehydrogenase), Ca^2+^‐dependent cellular processes and biomineralization (perlucin, neo‐calmodulin, and calmodulin), lysosomal protein degradation and immune response (lysozyme 3, cathepsins B and L1, tryptase‐2, mucin‐5 AC, integumentary mucin C.1), storage of soluble iron (ferritin lower subunit, soma ferritin, yolk ferritin, thioredoxin), protein disulfide isomerase, and homeostatic processes (apolipoprotein D and sonic hedgehog protein).

#### Turquoise

Ciliary activity (dynein inner arm light chain and dynein light chain roadblock‐type 2), unfolded protein response (eukaryotic translation initiation 3), molecular chaperones (prefoldin, T‐complex 1 subunits [alpha, beta, delta, zeta]), histones (i.e., H2A, H3, H4), free‐amino acid metabolism (carnosine synthase‐1), pH regulation (sodium/hydrogen exchanger 3), Ca^2+^‐dependent cellular processes (protein pif‐like, calmodulin 1, 2 and 3, neo‐calmodulin, and calmodulin striated‐muscle), cell detoxification (cytochrome P450), and regulation of oxidative stress (glutathione peroxidase 2 and metallothionein), pre‐mRNA splicing proteins, and transcription factors.

### 22‐day Oyster Spat

Each WGCNA module significantly correlated with experimental treatments was assessed for molecular functions and biological processes of gene groups. Genes associated with enriched pathways and GO terms included, but not limited to, the following proteins, protein families, and functions (parsed by module “color”):

#### Tan

Eppin, protease inhibitors (metalloproteinase, four‐domain, kunitz‐type, serine‐) and kielin‐chordin protein.

#### Turquoise

Carbonic anhydrase, molecular chaperones/cochaperones (dnaJ homologs, T‐complex protein subunits, cold shock domain‐containing protein, stress‐70, 10 kDa heat shock protein), sodium‐potassium pump (sodium/potassium‐transporting ATPase subunit alpha and beta‐3), free amino acid metabolism (alpha‐aminoadipic and methylmalonate semialdehyde dehydrogenases, aspartate aminotransferase) proteolysis (26S proteasome, E3 ubiquitin‐protein ligase TRIP12, ubiquitin‐like modifier‐activating enzyme 1), regulation of oxidative stress (peroxidase, peroxiredoxin, superoxide dismutase, peptidyl‐propyl cis‐trans isomerase), oxidative phosphorylation (citrate synthase, NADH dehydrogenase, cytochrome c oxidase, phosphoenolpyruvate carboxykinase, ATP synthase), glycolysis (malate dehydrogenase, pyruvate kinase PKM, fructose‐bisphosphate aldolase, and galactokinase), ribosomal biogenesis (39S and 40S), Ca^2+^‐dependent cellular processes and biomineralization (chitinase, caltractin, tyrosinase, calmodulin, calcium‐dependent protein kinase, calumenin, calcium‐transporting ATPase sarcoplasmic), ras‐related proteins, cargo transport and locomotary proteins (dynein intermediate chain [2 and 3], dynein light chain [2, roadblock‐type, and Tctex], dynein regulatory complex subunit 3, myosin‐2 essential light chain and myosin heavy chain, nonmuscle), signal transduction (i.e., serine/threonine protein kinases mig‐15, N2, PAK‐3), chromatin remodeling and histone lysine modifiers (i.e., histones H4, H2B, H1, and H2A, INO80 complex, mortality factor 4, histone‐lysine N‐methyltransferase), programmed cell death protein 5, pre‐mRNA processing, and splicing factors.

#### Green

Ca^2+^‐dependent cellular processes and biomineralization (perlucin, regucalcin, S‐crystallin, calmodulin striated‐muscle, calcium‐transporting ATPase sarcoplasmic), pH regulation (sodium‐bicarbonate cotransporter 3), protein degradation and immune response (cathepsin L1 and integumentary mucin C.1), and autophagy (beclin‐1, anti‐apoptotic protein NR13), unfolded protein response (activating transcription factor of chaperone, cyclic AMP‐responsive element‐binding protein 3, NF‐kappa‐B inhibitor‐like protein 1 and mitogen‐activated protein kinase 11), ribosomal proteins (60S, 40S), pre‐mRNA splicing (protein BUD31, serine/arginine‐rich splicing factor, small nuclear ribonucleoprotein F, splicing factor U2AF 50 kDa), oxidative phosphorylation (NADH dehydrogenase, cytochrome b‐c1 complex, cytochrome c oxidase), forkhead box protein K1, antioxidants (glutaredoxin, glutathione peroxidase 2), and homeostatic processes (5′‐AMP‐activated protein kinase and sonic hedgehog protein).

#### Blue

Ca^2+^‐dependent cellular processes and biomineralization (caltractin, tyrosinase, perlucin, regucalcin, and calmodulin A and 5), storage of soluble iron (soma ferritin, ferritin lower subunit), lysosomal protein degradation and immune response (cathepsin L and B, lysozyme 3), oxidative phosphorylation (NADH dehydrogenase and cytochrome b‐c1 complex subunits and c oxidase), carbohydrate metabolism/glycolysis (hexokinase), lysosomes, antioxidants (catalase, peroxiredoxin, glutathione peroxidase, glutaredoxin, metallothionein), catabolic processes (lipases, hydroxylases), immune response (tryptase‐2, mucin‐5 AC, chitotriosidase), and anticoagulation (antistasin, kappaPI‐antitoxin).

#### Red

Charged multivesicular body proteins (1a, 1b, 2a, 4b, 5), Ca^2+^‐dependent cellular processes and biomineralization (calcium homeostasis ER protein, and calcium/calmodulin‐dependent protein kinase type IV), carnosine synthase‐1, soma ferritin, cell detoxification (cytochrome P450), molecular chaperones/cochaperones (dnaJ homolog and hypoxia up‐regulated protein 1), transcription factors and regulators (transcription factor BTF3, transcription initiation factor TFIID, AP‐1, HES‐4, transcription initiation factors, TSC22 domain family protein, homeobox protein MSX‐3, Krueppel factor), ras‐related proteins (Rab 5B, 6, 7, and 9A), and immune response/apoptosis (apoptosis inducing factor‐3, eukaryotic translation initiation factor 1, NF‐kappa‐B inhibitor alpha).

#### Salmon

Calreticulin, unfolded protein response (activating transcription factor 3, eukaryotic translation initiation factor 2A, 2, and 3, 78 kDa glucose‐regulated protein, protein disulfide isomerases A3, A5, and A6), protein folding and molecular chaperones/cochaperones (T‐complex proteins, dnaJ homologs, heat shock proteins 90, 75, 10 kDa), ribosomal proteins (20S, 28S, 39S, and 50S), proteolysis (26S proteasome proteasome subunits alpha and beta), antioxidants (glutathione peroxidase, glutathione‐S transferase, peroxiredoxin‐5, peroxiredoxin), cellular locomotion (myosin regulatory light polypeptide 9 and unconventional myosin‐VI), carbonic anhydrase, TIM22 complex proteins (mitochondrial import inner membrane translocase subunits Tim10‐B, Tim13, and Tim22) S‐phase kinases, mitochondrial import receptors, acyl‐CoA dehydrogenase, carbonyl reductase, and apoptosis (apoptosis inducing factor‐1, programmed cell death protein 6, MAP kinase, and ras‐related proteins).
