# Supplementary material for: Transcriptome‐To‐Phenome Response of Larval Eastern Oysters Under Multiple Drivers of Aragonite Undersaturation
Source: Ecol Evol. 2025 Feb 11;15(2):e70953. doi: 10.1002/ece3.70953 (PMC11813985; doi:10.1002/ece3.70953)
Supplement: Supplementary file 1 — Data S1. [file ECE3-15-e70953-s001.docx]

**Supplementary Tables**

**Supplementary Table S1.** PERMANOVA model results for oyster larvae survival between 24 hpf and 15 dpf. Significant model effects are in bold (P < 0.05).


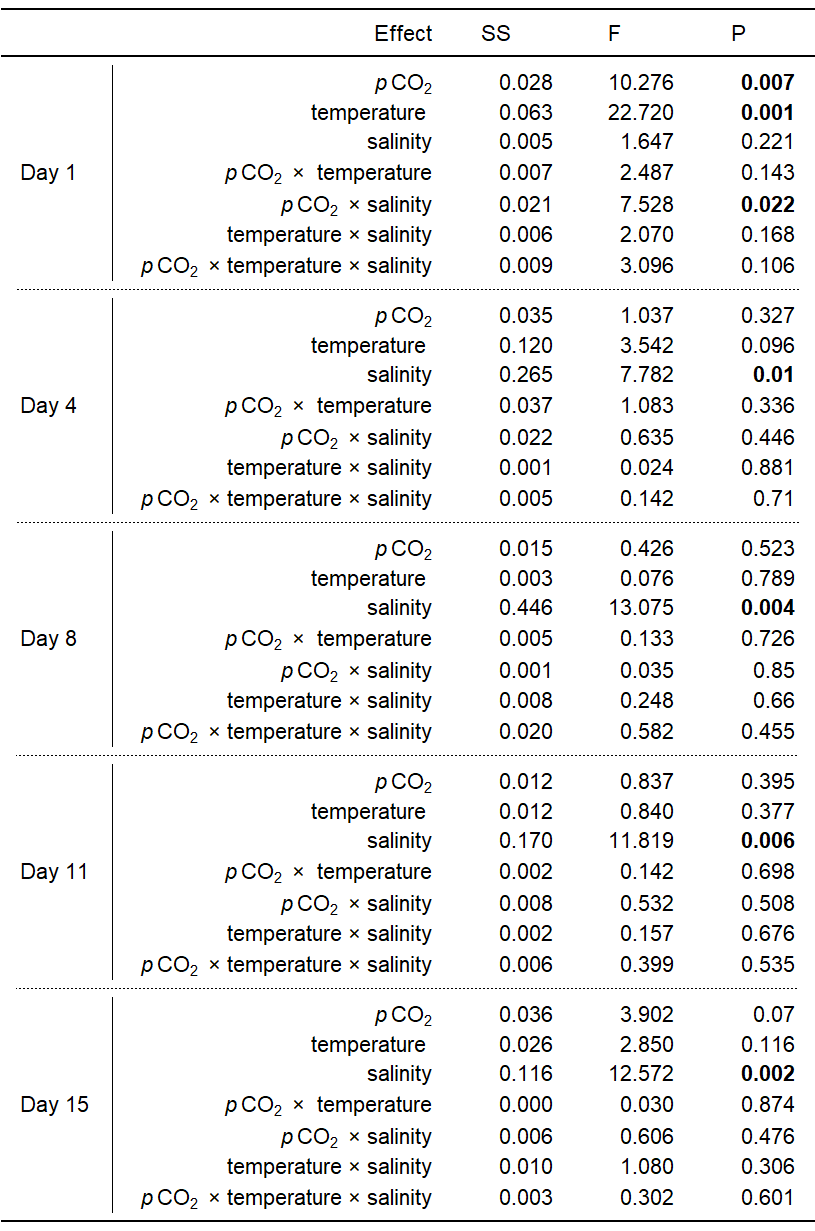


**Supplementary Table S2.** PERMANOVA model results for oyster larvae lengths between 24 hpf and 15 dpf. Significant model effects are in bold (P < 0.05).


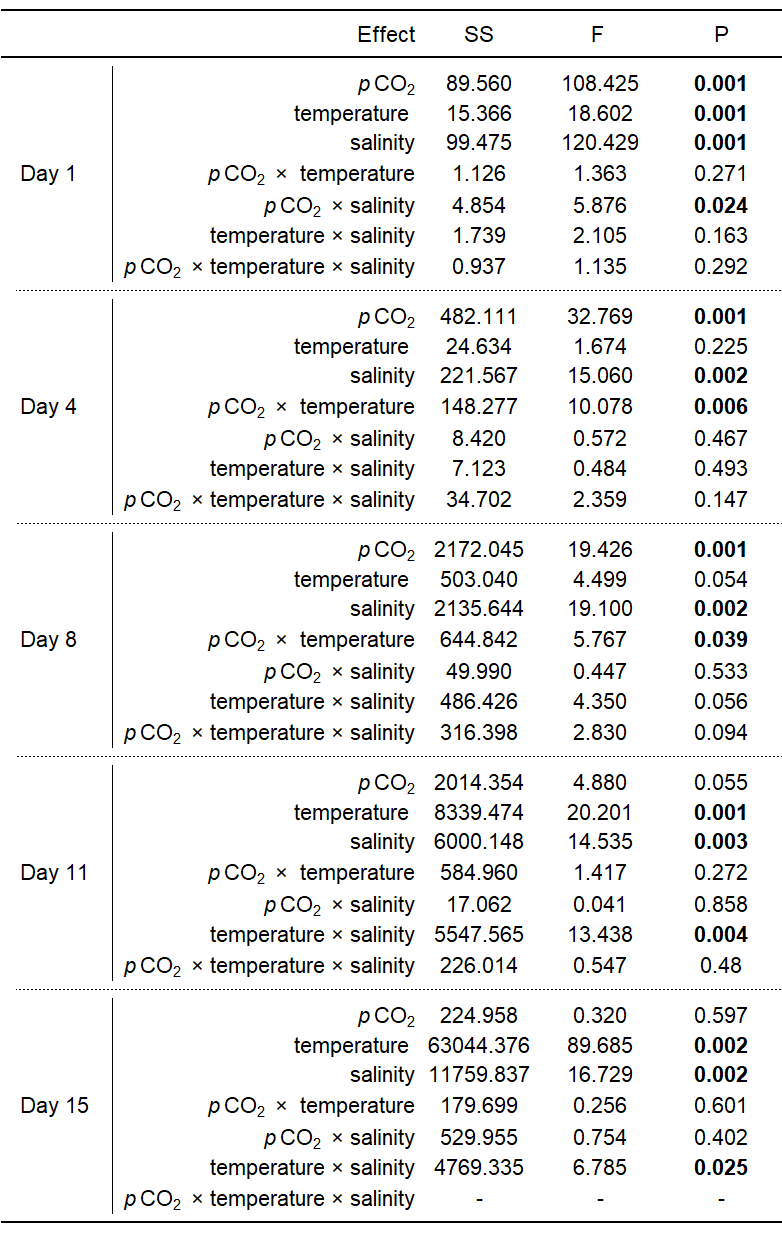


**Supplementary Table S3.** PERMANOVA model results for respiration rates of 24-hour oyster larvae. A significant model effect is in bold (P < 0.05).

**
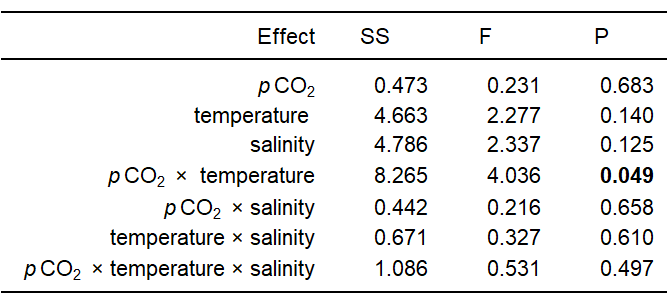
**

**Supplementary Table S4.** KEGG enrichment analysis of WGCNA data. Table is sorted alphabetically by module color.


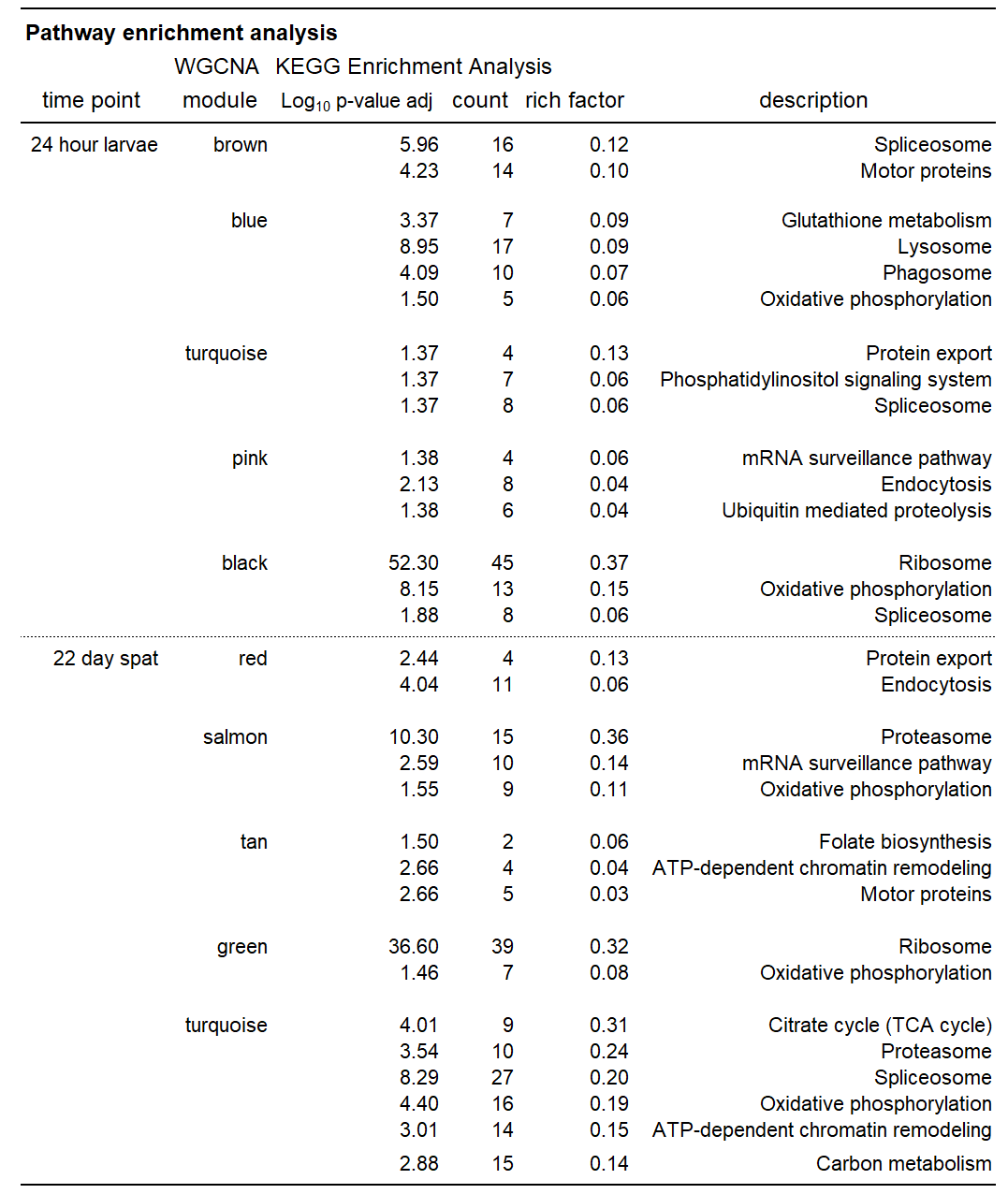


**Supplementary Figures**


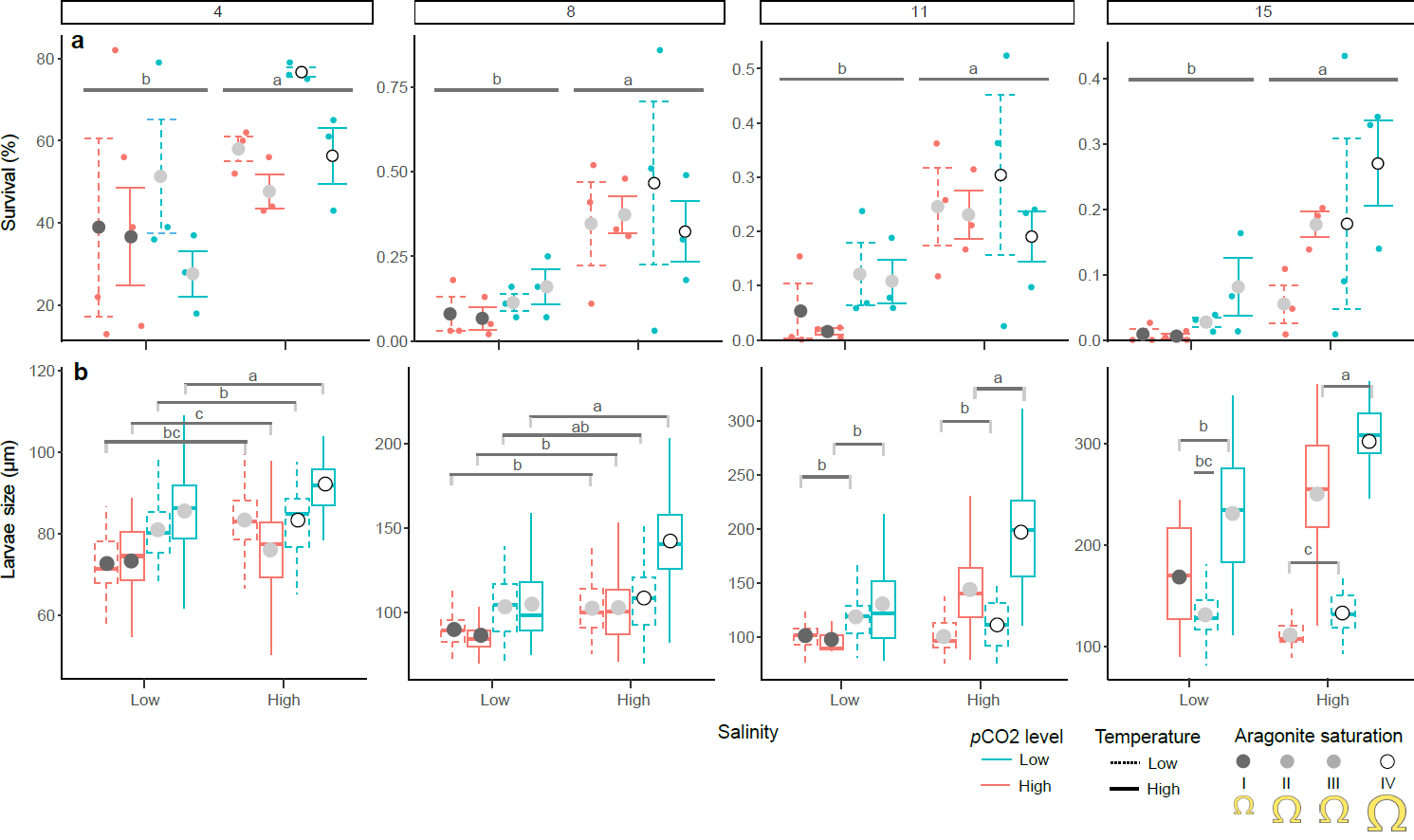


**Figure S1.** Survival (a) and length (b) of oyster larvae from 4 to 15 dpf; plots are organized by age in days. Survival is shown as mean ± SE (*N*=3 tanks treatment^-1^) and boxplots for length data with 25-75th percentile (boxes) and 1.5x interquartile range of all data (whiskers), mean (circles), and median length (horizontal line). Lower case letters represent pairwise differences from significant PERMANONA interaction terms.


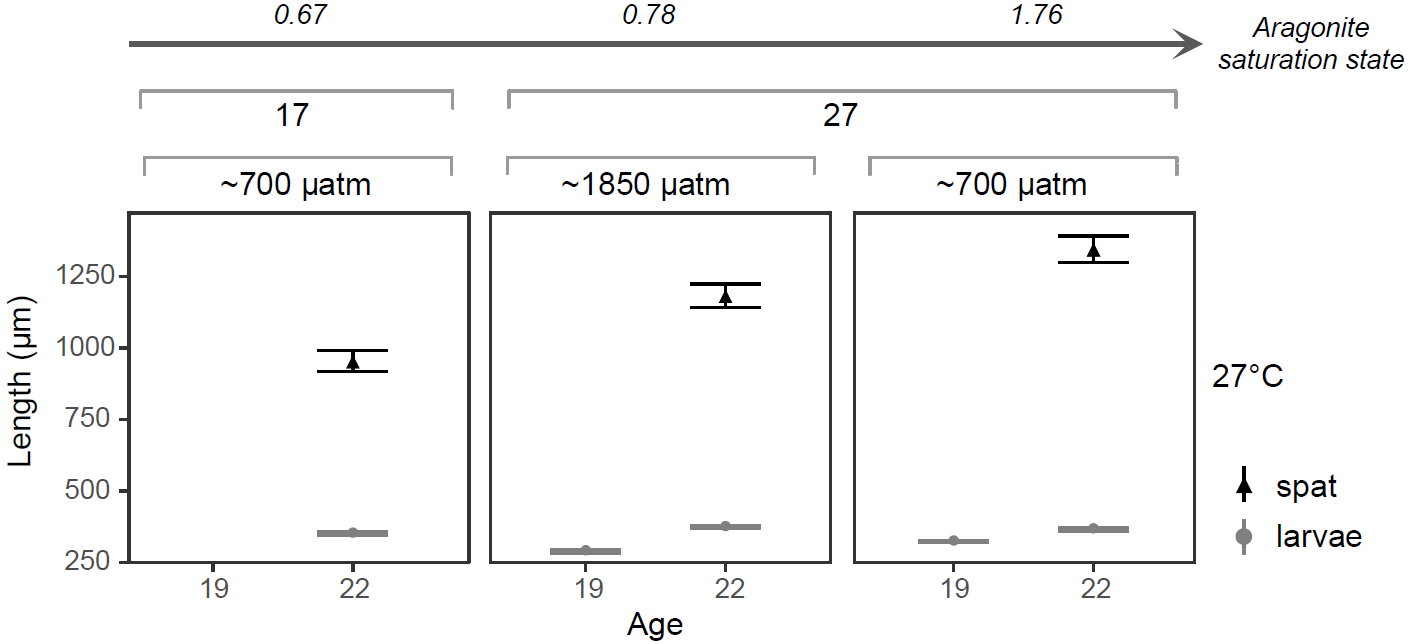


**Supplementary Figure S2.** Shell length (mean ± SEM) of oyster larvae and spat on days 19 and 22 post fertilization (mean ± SD; *N* = 20±8 and 45±25 larvae replicate tank^-1^). No spat or larvae lived to 19 dpf under low temperature and low Ω*ar*.


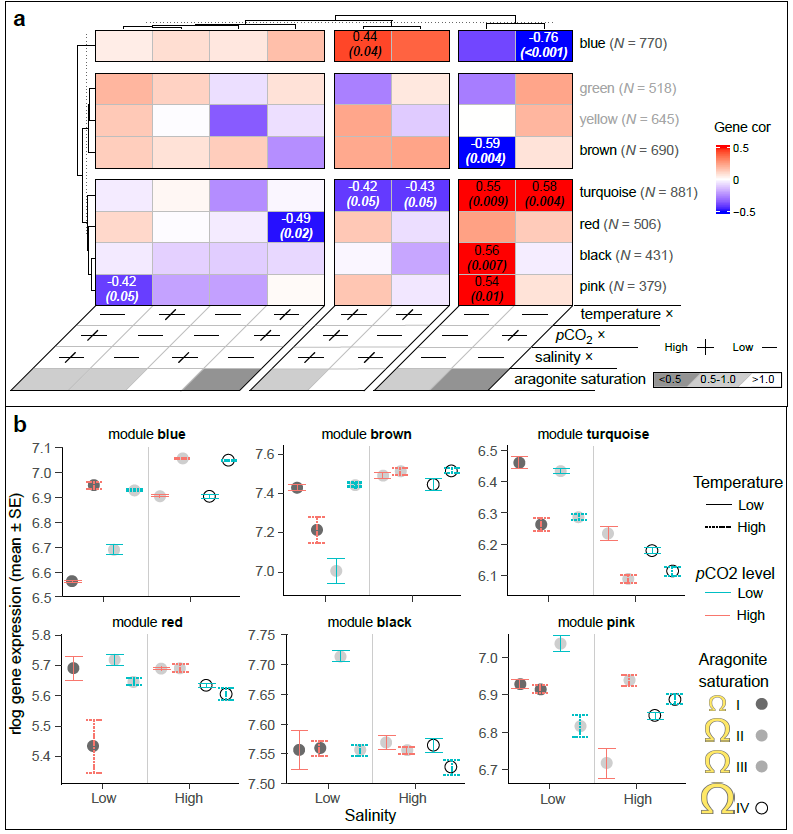


**Supplementary Figure S3.** WGCNA results for 24-hour larval oyster larvae. Heatmap represents gene-module correlations with temperature, salinity, and *p*CO_2_ treatment with the correlation coefficient and p-value shown for each significant module-treatment relationship (A); a module with an asterisk (i.e. module yellow) contains only main treatment correlation(s). rlog transformed gene expression data (mean ± SEM) is shown for all genes in significant modules (B; review A for *N* genes module^-1^), with data ordered by the four major treatment groups based on drivers of reduced aragonite saturation (roman numerals in module ‘blue’).


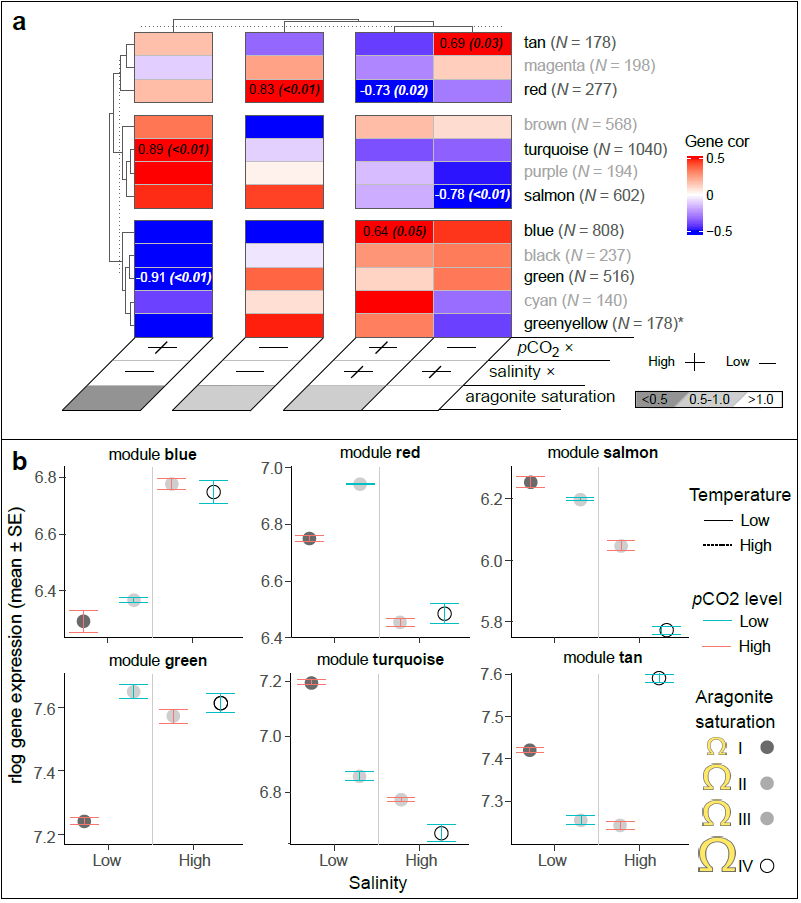


**Supplementary Figure S4.** WGCNA results for 22-day oyster spat, including only oysters under high temperature. Heatmap represents gene-module correlations with salinity and *p*CO_2_ treatment with the correlation coefficient and p-value shown for each significant module-treatment relationship (A); modules(s) with an asterisk (i.e. module pink) contain only main treatment correlation(s). rlog transformed gene expression data (mean ± SEM) is shown for all genes in significant modules (B; review A for *N* genes module^-1^), with data ordered by the four major treatment groups based drivers of reduced aragonite saturation (roman numeral in module ‘blue’).
